# Discovery of β-Arrestin-Biased 25CN-NBOH-Derived 5-HT_2A_ Receptor Agonists

**DOI:** 10.1021/acs.jmedchem.2c00702

**Published:** 2022-09-13

**Authors:** Christian B. M. Poulie, Eline Pottie, Icaro A. Simon, Kasper Harpsøe, Laura D’Andrea, Igor V. Komarov, David E. Gloriam, Anders A. Jensen, Christophe P. Stove, Jesper L. Kristensen

**Affiliations:** † Department of Drug Design and Pharmacology, Faculty of Health and Medical Sciences, University of Copenhagen, Universitetsparken 2, DK—2100 Copenhagen, Denmark; ‡ Laboratory of Toxicology, Department of Bioanalysis, Faculty of Pharmaceutical Sciences, Ghent University, Campus Heymans, Ottergemsesteenweg 460, B-9000 Ghent, Belgium; § Enamine Ltd., Kyiv 02094, Ukraine

## Abstract

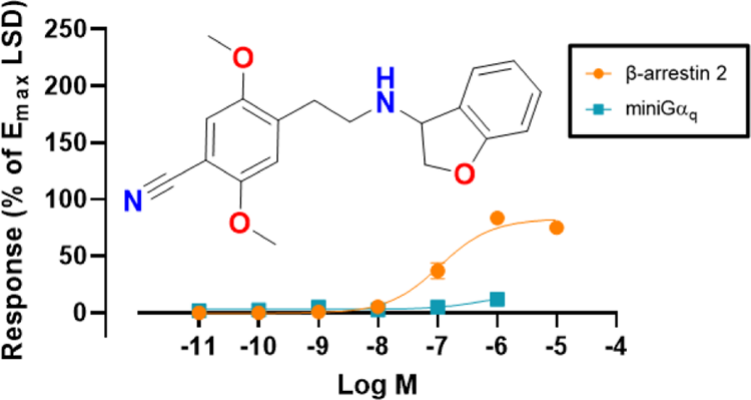

The serotonin 2A receptor (5-HT_2A_R) is the mediator of the psychedelic effects of serotonergic psychedelics, which have shown promising results in clinical studies for several neuropsychiatric indications. The 5-HT_2A_R is able to signal through the Gα_q_ and β-arrestin effector proteins, but it is currently not known how the different signaling pathways contribute to the therapeutic effects mediated by serotonergic psychedelics. In the present work, we have evaluated the subtype-selective 5-HT_2A_R agonist 25CN-NBOH and a series of close analogues for biased signaling at this receptor. These ligands were designed to evaluate the role of interactions with Ser159^3×36^. The lack of interaction between this hydroxyl moiety and Ser159^3×36^ resulted in detrimental effects on potency and efficacy in both βarr2 and miniGα_q_ recruitment assays. Remarkably, Gα_q_-mediated signaling was considerably more affected. This led to the development of the first efficacious βarr2-biased 5-HT_2A_R agonists **4a–b** and **6e–f**, βarr2 preferring, relative to lysergic acid diethylamide (LSD).

## Introduction

G protein-coupled receptors (GPCRs) form the largest protein family in the human genome, mediating signaling from the extracellular to the intracellular side of the cell membrane via a diverse range of neurotransmitters, hormones, and peptides.^1−3^ These transmembrane proteins are also the most prominent drug targets, with nearly one-third of all FDA-approved drugs acting on GPCRs. These receptors typically transduce physiological signals through intracellular G protein(s). Upon binding of an agonist, the heterotrimeric G protein interacts with the receptor, and the G_α_ subunit dissociates and initiates the downstream G-protein-mediated signaling cascade. Despite being the most prevalent targets in drug discovery campaigns, there have been limitations in understanding the *in vivo* pharmacological response of GPCR ligands through *in vitro* assays.^4,5^ Particularly the discovery of other effector proteins, such as β-arrestin 2 (βarr2), has shown the multifaceted nature of GPCR signaling.^6^ To fully untangle this complexity, pathway-selective (or biased) agonists need to be developed for each signaling pathway, i.e., ligands that lead to a preferential (ideally specific) activation of one of the alternative G protein and/or beta-arrestin signaling pathways.

The serotonin 2A receptor (5-HT_2A_R) is the most abundant excitatory serotonin receptor in the brain and the primary mediator of the psychedelic effects of serotonergic psychedelics. These psychedelics can be subdivided into three distinct chemotypes: ergolines, such as lysergic acid diethylamide (LSD),^7^ tryptamines such as psilocin^8^ (which was first isolated from *Psilocybe Mexicana*),^9^ and phenylalkylamines, such as 2,5-dimethoxy-4-iodoamphetamine (DOI)^10^ and mescaline^11,12^ (which was first isolated from *Lophophora williamsii*).^13^ In recent years, there has been increased scientific interest in these serotonergic psychedelics primarily based on the work with psilocybin, which has displayed promising effects in clinical studies focused on various neuropsychiatric indications, including depression and anxiety,^14−17^ substance abuse,^18−20^ and obsessive-compulsive disorder (OCD).^21^ Besides psilocybin, there has also been a renewed interest in the medical use of LSD.^22,23^ In addition to its 5-HT_2A_R agonism, psilocybin exhibits high agonist potency at most of the serotonergic receptors^24,25^ and LSD possesses high activity at an even broader range of monoaminergic receptors.^26^ Despite these nonselective receptor profiles, the activation of 5-HT_2A_R is considered essential for the psychedelic effects as well as the apparent therapeutic potential of these compounds. In general, the phenylalkylamines, and in particular *N*-benzylphenethylamines (NBOMe’s), have shown selectivity toward 5-HT_2A_R. The reader is referred to recent review articles for a more in-depth discussion on the historical overview of the NBOMe class.^27,28^ Despite efforts from many, the success rate of developing truly selective 5-HT_2A_R agonists has been nominal.^29−31^ The most notable exceptions are 25CN-NBOH^32−35^ and (*S,S*)-DMBMPP,^36^ which are the most selective 5-HT_2A_R agonists reported to date, with a 52- to 100-fold and 124-fold selectivity over 5-HT_2C_R, respectively.^33,36^

The 5-HT_2A_R is able to signal through members of both Gα_q_ and Gα_i/o_ protein families and also through beta-arrestin mediated pathways.^37,38^ Several psychedelics and 5-HT_2A_R agonists have been evaluated for their respective bias profiles toward the Gα_q_ and βarr2 transducers, by means of highly analogous functional complementation assays.^39−42^ Recently, the first partial agonist (*E*_max_ = 13%) with bias toward the βarr2 over Gα_q-γ9_ pathway, compared to the reference 5-HT, has been disclosed (IHCH-7086).^43^ However, no strongly biased agonist for the G-protein-mediated signaling pathway has been identified. Herein, we have profiled the subtype-selective agonist 25CN-NBOH and a series of close analogues for functional selectivity at 5-HT_2A_R in Gα_q_- and βarr2-based functional assays and have evaluated the role of the simultaneous interaction of Ser159^3×36^ with the ammonium and the benzylic hydroxyl of the ligands,^44^ which led to the discovery of the first efficacious βarr2-biased agonists for this receptor, relative to LSD.

## Results and Discussion

### Chemistry

**Scheme 1:**
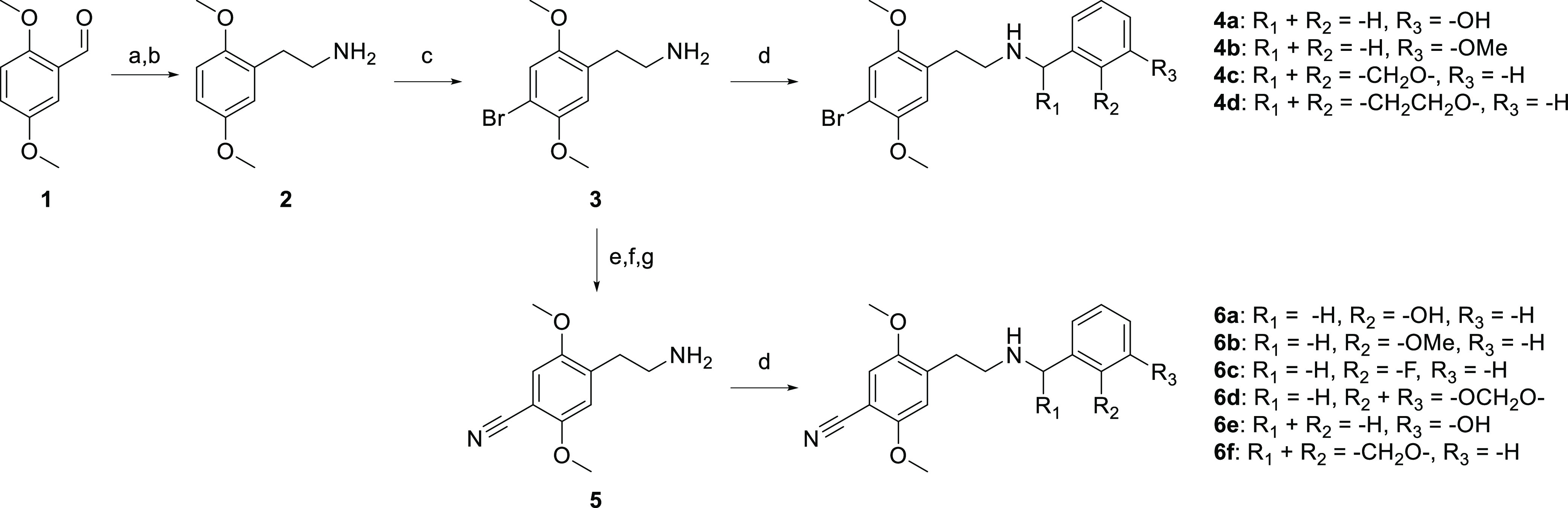
Synthesis of *N*-Benzylphenethylamines Reaction conditions: (a) nitromethane, NH_4_OAc, 100 °C; (b) LAH, tetrahydrofuran (THF) reflux; (c) Br_2_, AcOH, rt; (d) aldehyde, EtOH, rt or ketone, AcOH, MeOH/THF, rt; (ii) NaBH_4_, EtOH, rt or NaBH_3_CN, THF, rt; (e) phthalic anhydride, toluene, reflux; (f) Cu(I)CN, *N*,*N*-dimethylformamide (DMF), reflux; (g) hydrazine (aq.), THF, rt.

The corresponding phenethylamine analogues were all prepared according to previously described methods.^10,32,45,46^ In short, aldehyde **1** was condensed with nitromethane and subsequently reduced with lithium aluminum hydride (LAH), to yield phenethylamine **2** (2C-H) (Scheme 1). Compound **3** (2C-B) was prepared via subsequent bromination of the 4-position.^10^ The corresponding *N*-benzyl derivatives (**4a–b**) were prepared via reduction amination of **3**, in the presence of the appropriate benzylaldehyde.^45^ Analogues **4c–d** were prepared from condensation of **3** with 3-coumaranone or 4-chromanone, respectively. The resulting imines were reduced with NaBH_3_CN, which yielded the racemic secondary amines in 52–58% isolated yield (Scheme 1). To obtain phenethylamine **5** (2C-CN), compound **3** was converted to the corresponding phthalimide. Subsequent copper-catalyzed cyanation on the 4-bromo moiety and the phthalimide deprotection with NH_2_NH_2_^46^ led to **5**, from which the corresponding *N*-benzyl derivatives (**6a–e**) were prepared via reduction amination in the presence of the appropriate benzylaldehyde.^32,45^**6f** was prepared from condensation of **5** with 3-coumaranone. The resulting imine was reduced with NaBH_3_CN, which yielded the racemic secondary amine in 55% (Scheme 1).

### Pharmacological Characterization

The functional characteristics of **4a–d** and **6a–f** at the 5-HT_2A_R were determined by bioassays using the Nanoluciferase Binary Technology (NanoBiT).^40,41^ Briefly, the two nonfunctional parts of the nanoluciferase are each fused to one of the two interacting proteins, in this case the 5-HT_2A_R and the cytosolic proteins, βarr2 or miniGα_q_, i.e., the GTPase domain of the Gα_q_ subunit.^47−49^ Upon receptor activation, the cytosolic proteins are recruited to the intracellular parts of the receptor, leading to the functional complementation of the split-nanoluciferase and generation of a luminescent signal, in the presence of the enzyme’s substrate.^50^ Both the potency and efficacy of the evaluated compounds were determined with this setup. To allow the comparison of the obtained results with previous results, LSD was chosen as the reference agonist for *E*_max_ and β-factor calculations, and serotonin (5-HT) was included as a positive control.^41^ The functional data normalized to 5-HT as a reference agonist is included in the Supporting Material (Table S1). To obtain the data given in Table 1, the area under the curve (AUC) of the full (standard) 2 h activation (time–luminescence) profiles was used to generate concentration–response curves. For a more detailed comparison of biased agonism of (psychedelic) phenethylamines with various incubation times, the reader is referred to Pottie and Poulie et al.^51^

**Table 1:** Functional Properties of the Compounds (**4a–d** and **6a–f**) at 5-HT_2A_R in the βarr2 or miniGα_q_ Recruitment Assays^a^

	β-arr2		miniGα_q_		
5-HT_2A_	EC_50_ (nM) [CI]	*E*_max_ (%) [CI]	EC_50_ (nM) [CI]	*E*_max_ (%) [CI]	β-factor
5-HT	12.1 [8.52–17.4]	110 [105–115]	130 [63.3–270]	222 [197–249]	0.576
LSD	12.9 [8.45–19.7]	99.7 [93.6–106]	13.2 [6.81–25.6]	100 [91.0–110]	0
**4a**	11.1 [7.65–16.2]	112 [105–118]	48.8 [13.0–157]	28.0 [22.1–34.7]	1.240
**4b**	11.1 [7.59–16.3]	113 [106–120]	44.4 [19.1–94.6]	38.8 [34.0–44.3]	1.100
(±)-**4c**	28.6 [18.9–43.3]	96.6 [90.4–103]	23.0 [12.0–44.6]	48.7 [44.0–53.7]	0.279
(±)-**4d**	132 [108–161]	121 [115–126]	174 [80.9–423]	47.7 [40.4–57.5]	0.558
**6a** (25CN-NBOH)	2.75 [1.73–4.40]	150 [141–160]	8.59 [3.87–18.1]	123 [110–136]	0.619
**6b** (25CN-NBOMe)	1.93 [1.17–3.28]	161 [151–171]	6.71 [3.82–11.4]	159 [148–170]	0.526
**6c** (25CN-NBF)	53.2 [36.8–75.7]	114 [107–121]	168 [77.7–363]	72.5 [62.8–82.9]	0.669
**6d** (25CN-NBMD)	17.0 [10.4–28.2]	114 [106–123]	45.1 [14.5–128]	83.0 [67.1–100]	0.638
**6e**	84.5 [64.0–111]	106 [101–112]	301 [46.1–1764]	22.5 [16.4–30.3]	1.250
(±)-**6f**	108 [68.6–169]	82.9 [75.9–90.2]	631 [n.d.]	18.0 [n.d.]	n.d.

a Data obtained in the βarr2 or miniGα_q_ recruitment assays, using the 2 h time–luminescence profile to calculate the AUC. The EC_50_ value is a measure of agonist potency, and the *E*_max_ value is a measure of agonist efficacy. The *E*_max_ values for the compounds are normalized to the *E*_max_ of LSD as the reference agonist (data for the compounds where the *E*_max_ are normalized to serotonin *E*_max_ values can be found in the Supporting Information). Data are combined from at least three independent experiments, each performed in duplicate. The reported β-factor is the average value of the three β-factors obtained in three independent experiments; β-factors derived from the “combined” EC_50_ and the *E*_max_ values can be found in Table S2. n.d. is not determined; see text for further details. CI: 95% confidence interval.

**Figure 1:**
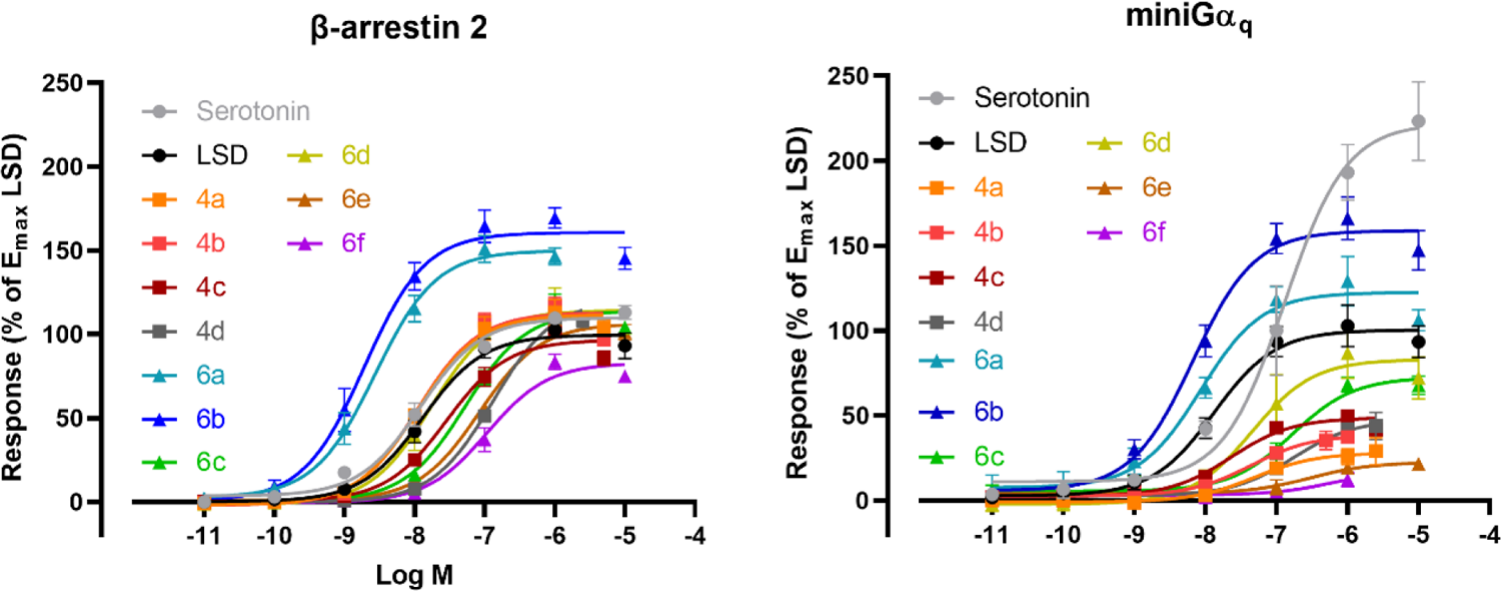
Concentration–response curves of the tested compounds (**4a–d**, and **6a–f**) at the 5-HT_2A_R in the βarr2 or miniGα_q_ recruitment assays. Overlay of the concentration–response curves for each of the tested substances in the two assay formats. The *E*_max_ values for the compounds are normalized to *E*_max_ of LSD as the reference agonist (data for the compounds where *E*_max_ are normalized to serotonin *E*_max_ values can be found in the Supporting Information). Each point represents the mean of three independent experiments, each performed in duplicate ± standard error of the mean (SEM). Curves represent three parametric, nonlinear fits.

**Figure 2:**
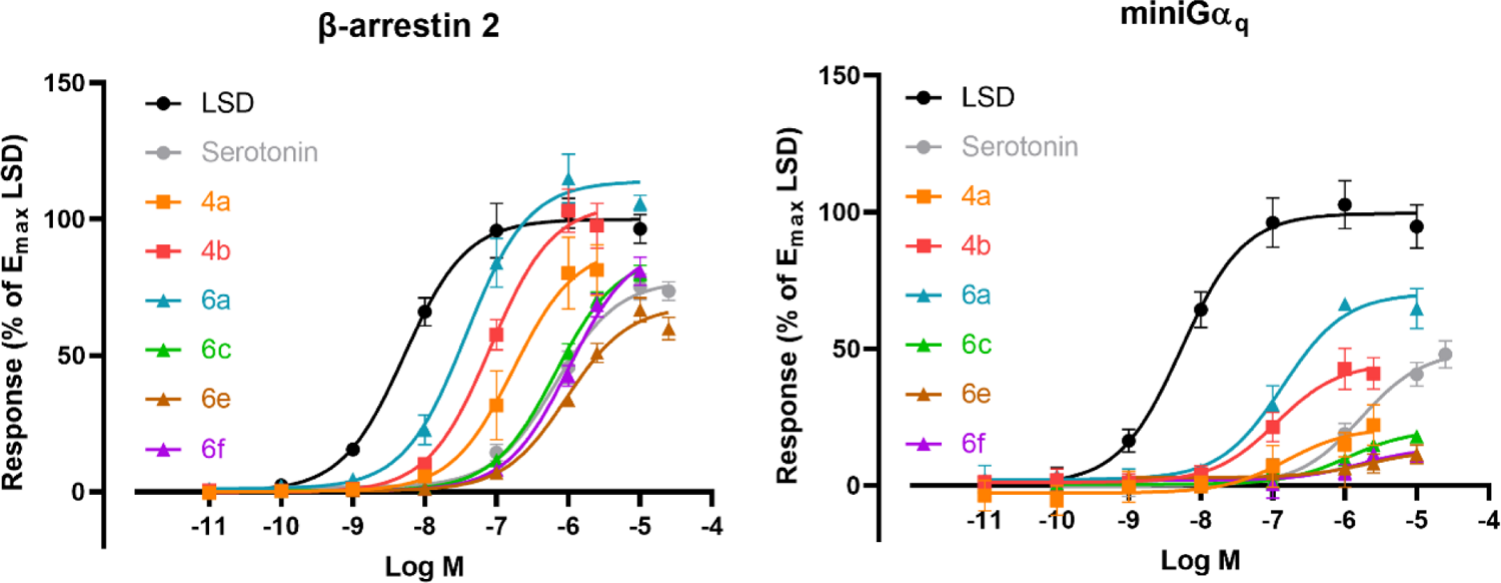
Concentration–response curves of the compounds (**4a–b** and **6a,c,e–f**) at the 5-HT_2A_R- S159A mutated receptor in the βarr2 or miniGα_q_ recruitment assays. Overlay of the concentration–response curves for each of the tested substances in the two assay formats. The *E*_max_ values for the compounds are normalized to *E*_max_ of LSD as the reference agonist (data for the compounds where *E*_max_ are normalized to serotonin *E*_max_ values can be found in the Supporting Information). Each point represents the mean of three independent experiments, each performed in duplicate ± standard error of the mean (SEM). Curves represent three parametric, nonlinear fits.

**Figure 3:**
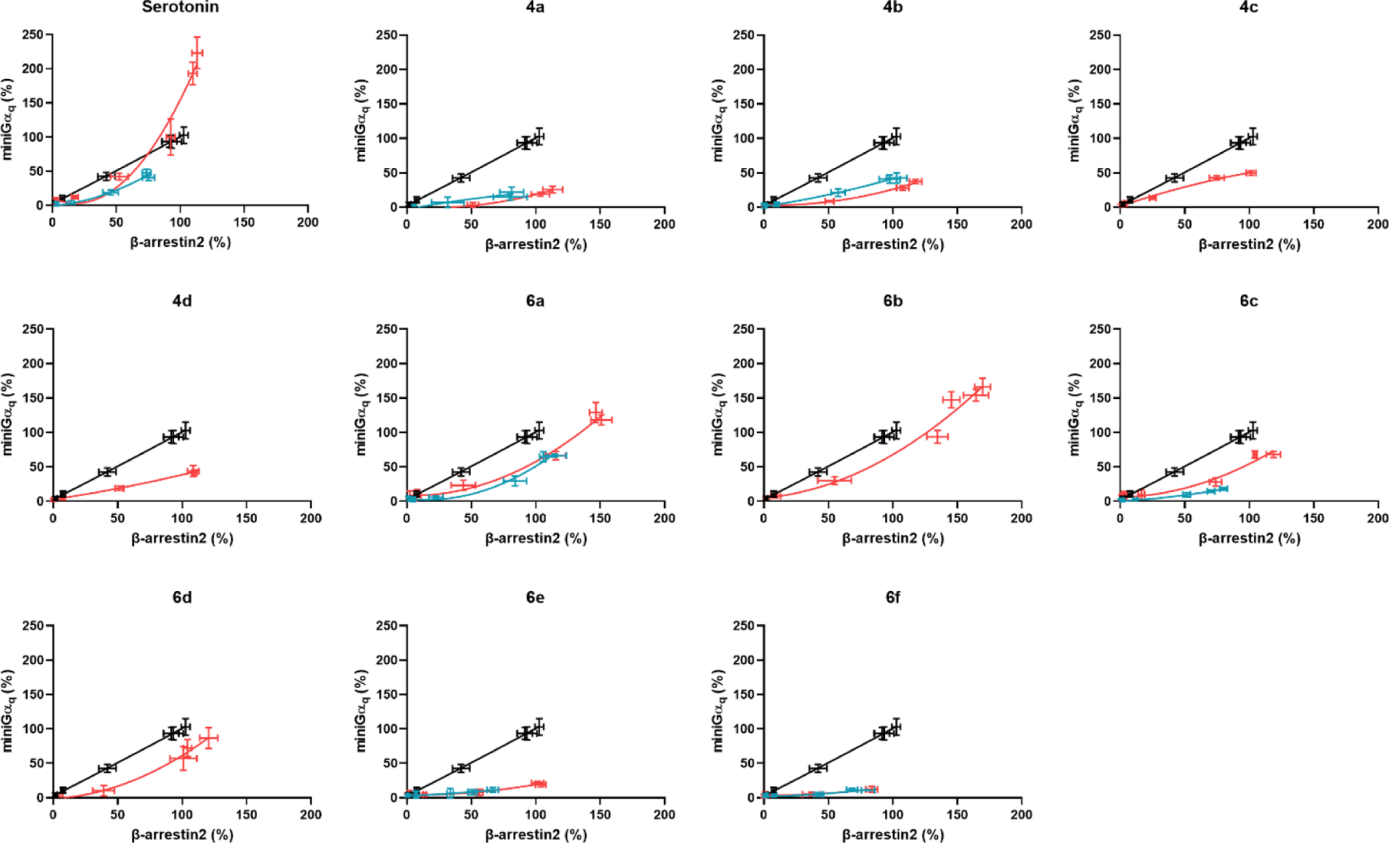
Qualitative bias plots, where each panel shows the centered second-order polynomial fit of the activation values at equimolar concentrations of the substance in the respective assays in red, and that of the reference agonist (LSD) in black (WT and S159A mutated receptor overlap for LSD). Red is data for WT receptor, and blue is data for S159A mutated receptor. Error bars represent the SEM of the individual data points per concentration.

**Figure 4:**
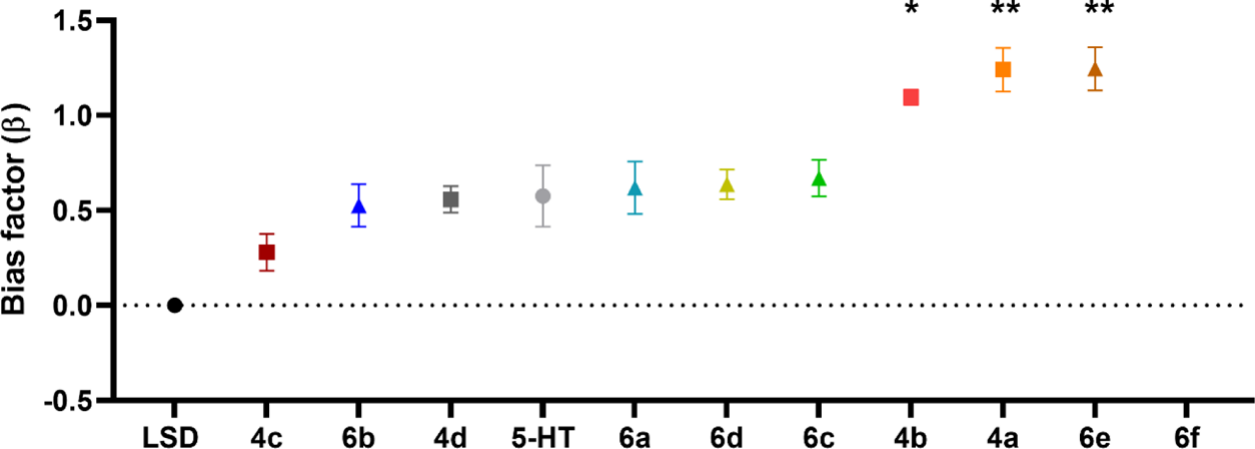
Visual representation of the bias factors (β), where * stands for *p* < 0.05 and ** stands for *p* < 0.01 in the nonparametric Kruskal–Wallis analysis of significance. Compound **6f** is omitted, as no bias factor could be calculated. LSD is used as the reference agonist (data for the compounds where serotonin is used as the reference agonist can be found in the Supporting Information).

The EC_50_ and *E*_max_ values (normalized to *E*_max_ of LSD), as a measure of potency and efficacy, respectively, for the compounds are summarized in Table 1. Additionally, the EC_50_ and *E*_max_ values of compounds **4a–b** and **6a,c,e–f** were also determined at the 5-HT_2A_R S159A-mutated receptor, and these data are summarized in Table 2. The S159A residue was mutated because of its double interaction with 25CN-NBOH (**6a**) in the deposited cryo-EM structure: Ser159 interacts simultaneously with both its ammonium and its *ortho*-OH moiety on the benzyl ring.^52^ Additionally, Kim et al.^52^ previously reported that **6a** and serotonin show 161- and 157-fold decreases in potency in a Gα_q_-dissociation BRET assay at 5-HT_2A_R, respectively, with the introduction of the S159A mutation, while the efficacy of the two agonists remained roughly unchanged for serotonin and decreased by a quarter, for **6a**. Most of the evaluated ligands lack the possibility to interact with Ser159^3×36^, making it compelling to investigate the influence of this residue on the biased agonism of these ligands. The agonist concentration–response curves of all compounds are presented in Figure 1A (βarr2) and Figure 1B (miniGα_q_) for the wild-type receptor, and Figure 2A,B, respectively, for the S159A mutated receptor. Figure 3 illustrates the bias plots of the respective ligands evaluated, and Figure 4 shows the overview of the Kruskal–Wallis analysis of the bias factors. These data with serotonin as reference can be found in Figures S2–6 and Table S1, in the Supporting Information.

**Table 2:** Functional Properties of the Tested Compounds (**4a–b** and **6a, c, e–f**) at the 5-HT_2A_R S159A-Mutated Receptor in the βarr2 or miniGα_q_ Recruitment Assays^a^

	β-arr2		miniGα_q_		
5-HT_2A_-S159A	EC_50_ (nM) [CI]	*E*_max_ (%) [CI]	EC_50_ (nM) [CI]	*E*_max_ (%) [CI)	β-factor
5-HT	661 [415–1025]	77.4 [71.7–83.4]	1672 [728–4550]	49.3 [41.3–60.4]	0.550
LSD	5.19 [3.20–8.25]	99.9 [93.7–106]	5.38 [2.85–9.81]	99.5 [91.6–108]	0
**4a**	172 [74.8–443]	89.8 [75.8–108]	154 [n.d.]	21.0 [n.d.]	n.d.
**4b**	81.9 [52.6–126]	106 [97.5–114]	112 [40.1–327]	44.7 [36.9–53.9]	0.565
**6a** (25CN-NBOH)	37.7 [23.3–59.9]	114 [105–123]	137 [76.2–247]	70.3 [62.2–78.9]	0.733
**6c** (25CN-NBF)	661 [504–853]	86.6 [81.5–92.0]	1126 [253–3869]	20.4 [14.1–30.3]	0.731
**6e**	939 [614–1388]	68.2 [62.6–74.3]	2064 [n.d.]	12.9 [n.d.]	n.d.
(±)-**6f**	1025 [673–1517]	90.8 [81.9–101]	1653 [n.d.]	13.8 [n.d.]	n.d.

a Data obtained in the βarr2 or miniGα_q_ recruitment assays, using the 2 h time–luminescence profile to calculate the AUC. The EC_50_ value is a measure of agonist potency, and the *E*_max_ value is a measure of agonist efficacy. The *E*_max_ values for the compounds are normalized to *E*_max_ of LSD as the reference agonist (data for the compounds where *E*_max_ are normalized to serotonin *E*_max_ values can be found in the Supporting Information). Data are combined from at least three independent experiments, each performed in duplicate. The reported β-factor is the average value of the three β-factors obtained in three independent experiments; β-factors derived from the “combined” EC_50_ and *E*_max_ values can be found in Table S2. n.d. is not determined; see text for further details. CI: 95% confidence interval.

The 4-bromo analogues (**4a–c**) displayed nanomolar agonist potency at 5-HT_2A_R in both the βarr2 (EC_50_: 11–29 nM) and the miniGα_q_ (EC_50_: 23–49 nM) recruitment assays, which is in line with previously reported values for 4-halogen-substituted analogues, such as 25I-NBOMe and 25I-NBOH.^41^ Interestingly, the *E*_max_ values exhibited by these analogues were reduced compared to 25I-NBOMe and 25I-NBOH, albeit not as pronounced in the βarr2 assay (*E*_max_: 97–113% vs 135–141%, respectively) as in the miniGα_q_ assay (*E*_max_: 28–49% vs 111–160%, respectively) (Table 1). Interestingly, extension of the dihydrobenzofuran ring of **4c** with one carbon markedly reduced the potency of **4d** in both the βarr2 and miniGα_q_ recruitment assays (EC_50_: 132 and 174 nM, respectively) compared to that of **4c**, whereas this modification had little influence on the agonist efficacies in either assay (121 and 48%, respectively) compared to **4a–c**. Despite minor variations in the potencies and efficacies of the four bromo analogues (**4a–d**), there was a marked difference in their calculated β-factor. For example, **4a–b** were statistically significant βarr2-preferring agonists, with β-factors of 1.24 and 1.10, respectively (Table 1), relative to LSD in contrast to **4c** with a β-factor of 0.279, which is in line with most other NBOMes.^41^

The 4-cyano analogues (**6a–f**) displayed more mixed potency profiles compared to the 4-bromo analogues (**4a–d**). Compounds **6a–b** displayed agonist potencies in the low nanomolar range at 5-HT_2A_R in both the βarr2- (EC_50_: 2.8 and 1.9 nM, respectively) and the miniGα_q_-recruitment assays (EC_50_: 8.6 and 6.7 nM), with *E*_max_ values of 150 and 161% in the βarr2-assay and 123 and 159% in the miniGα_q_-assay, respectively. This is in line with the reported values for 25H-NBOH and 25H-NBOMe.^41,42^ Compound **6c** followed the same trend as **6a–b**, albeit with significantly lower potencies and efficacies at the receptor for the recruitment of both cytosolic mediators. Interestingly, **6d** displayed reduced potencies and efficacies in the βarr2 and miniGα_q_ assays compared to those of **6a–b**, but both were increased or the same compared to **6c**, respectively. This tendency is similar to what is observed with the 4-bromo analogues (**4a–b**), which also lack a hydrogen-bond acceptor in the ortho-position on the benzylic ring.

Compounds **6a–d** displayed slightly lower *E*_max_ values in the miniGα_q_ than in the βarr2 recruitment assay, and interestingly, the efficacies displayed by the other compounds in the miniGα_q_ recruitment assay were only half or even lower than the corresponding efficacies in the βarr2 assay compared to other NBOMes.^41^ This resulted in a particularly strong preference toward βarr2 recruitment for **4a–b** and **6e** with calculated β-factors ranging 1.10–1.25, relative to LSD. While no β-factor for **6f** could be calculated because of its low activity in the miniG_αq_ recruitment assay, judging from the bias plot (Figure 3), it is apparent that this ligand was highly biased for βarr2 recruitment, relative to LSD Table S2. This observation is numerically reflected when using a slightly different method of data analysis, as shown in Supplementary Table S2.

Of note, even though the obtained absolute bias factors are different when serotonin is taken as the reference agonist (Table S1 and Figure S6), these three compounds still show a preference toward βarr2 recruitment relative to serotonin. From the bias plot (Figure S5) of compound **6f**, also a strong preference toward βarr2 recruitment relative to serotonin can be deduced. For a more detailed comparison of biased agonism of (psychedelic) phenethylamines relative to reference agonists LSD and serotonin, the reader is referred to Pottie and Poulie et al.^51^

Regarding the S159A mutated 5-HT_2A_R, it should first be noted that serotonin displayed a significant loss of potency and efficacy at this mutated receptor compared to the WT receptor in both the βarr2 and miniGα_q_ recruitment assays. The fact that the agonist potency of LSD at 5-HT_2A_R was not affected by this mutation prompted us to use LSD as the reference agonist to enable comparisons between the WT and mutated receptor (Table 2 and Figure S8). The potency of **4a** was reduced at 5-HT_2A_R S159A compared to WT 5-HT_2A_R by factors of approximately 15 and 3 in the βarr2 and miniGα_q_ recruitment assays, respectively. On the other hand, agonist potency of **4b** in the βarr2 assay was only negatively affected by a factor of 7, which is to be expected from the loss of a hydrogen-bond interaction. Remarkably, the efficacy of **4b** remained largely unaffected by the S159A mutation, as neither its potency nor its efficacy in the miniGα_q_ recruitment assay was significantly altered. The agonist potency displayed by **6a** at the S159A mutated 5-HT_2A_R in the βarr2 recruitment assay was likewise reduced (approximately 14-fold) as it has been reported previously.^52^ In this case, the efficacy was also considerably decreased (*E*_max_: 150 and 114% at WT 5-HT_2A_R and 5-HT_2A_R S159A, respectively). The same was observed in the miniGα_q_ recruitment assay, with substantially reduced agonist potency and a significant decrease in efficacy (EC_50_: 8.6 and 137 nM, *E*_max_: 123 and 70% at WT 5-HT_2A_R and 5-HT_2A_R S159A, respectively). The agonist potencies displayed by **6c** at 5-HT_2A_R S159A were reduced by factors of 12 and 7 at the mutated receptor compared to the WT receptor in the βarr2 and miniGα_q_ recruitment assays, respectively, and its agonist efficacies also decreased substantially by the introduction of the mutation (Table 2). The agonist potencies of **6e–f** also decreased by factors of ∼10 at the S159A mutated receptor in the βarr2 recruitment assay, and the potency of **6e** at the mutated receptor in the miniGα_q_ recruitment assay was more affected compared to that of **6f** (6.9-fold compared to 2.6-fold, respectively). Of note is that, although all experiments with the mutated receptor were conducted relative to reference agonists, we cannot fully exclude that different expression levels of the wild-type and mutated receptor constructs may have some impact.

Taken together, from these results it is apparent that regardless of the benzylic substituent, the 4-bromo analogues and the 4-cyano analogues do not exhibit the same structure–activity relationship (SAR). In particular, this is highlighted in the clear difference in the relative preference exhibited by these two analogue series when it comes to βarr2 recruitment to the 5-HT_2A_R (Figure 1 and Table 1). An exception to this is the fact that **4a** and **6e** display the same trend (β-factors of 1.240 and 1.250, respectively). Furthermore, the change of the benzylic hydroxy in the ortho-position in **6a**, to the meta-position in **6e**, resulted in a significant loss of both agonist potency and efficacy in both the βarr2 and miniGα_q_ recruitment assays. However, the efficacy was more significantly reduced in the miniGα_q_ assay, which resulted in a β-factor signifying a stronger preference toward βarr2 recruitment for **6e**. This suggests that, at least for the 4-cyano analogues, this interaction with Ser159^3×36^ is desired for the recruitment of miniGα_q_.^32,52^

### Binding Mode Analysis

**Figure 5:**
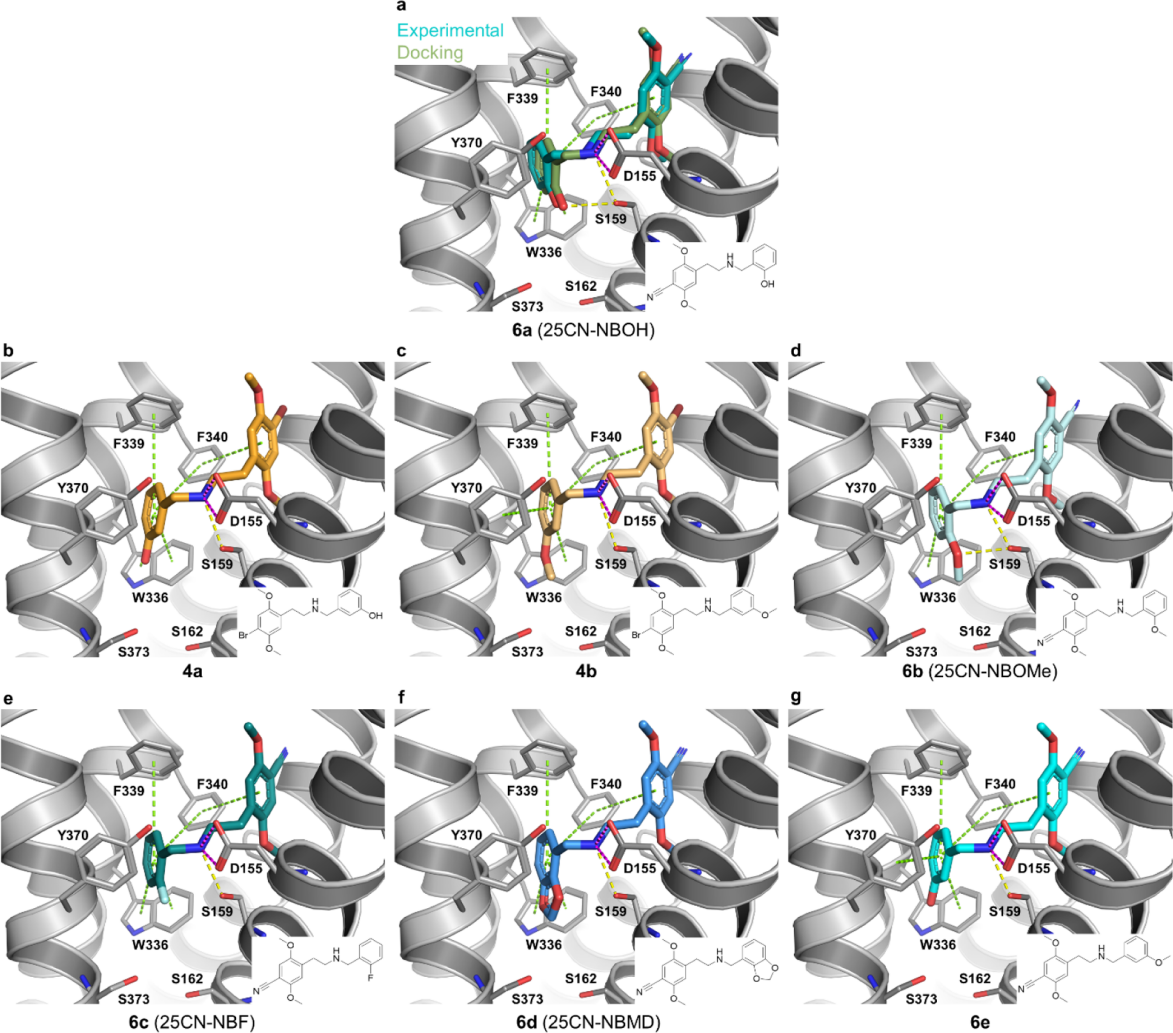
Experimental and predicted binding modes of **4a–b** and **6a–e** to the 5-HT_2A_R. (A) Comparison between the experimental (cyan) and the redocking binding pose of **6a** in the cryo-EM structure of the G_q_-coupled 5-HT_2A_R (PDB ID 6WHA)^52^ (RMSD of 0.57 Å for heavy atoms). (B–G) Predicted binding poses and ligand–receptor interactions for **4a–b** and **6b–e** to the 5-HT_2A_R. The ligands are displayed as sticks, while the receptor is shown as gray lines and cartoon. Ligand–receptor interactions are displayed as dashed lines and colored in green (aromatic, π–π staking), yellow (hydrogen bond), and pink (salt-bridge).

**Figure 6:**
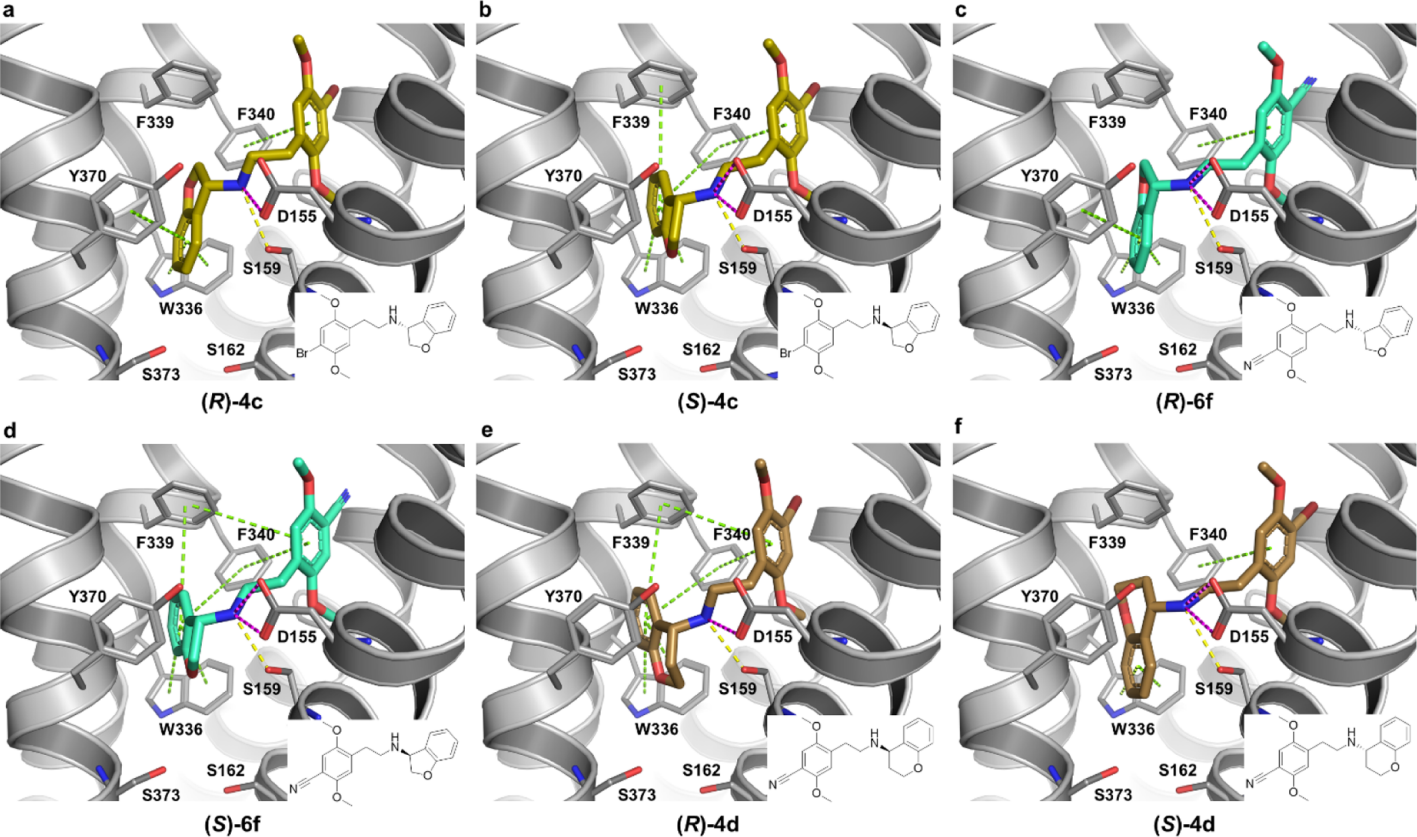
Predicted binding mode of both enantiomers of **4c–d** and **6f** to the 5-HT_2A_R. A–F. Predicted binding poses and ligand–receptor interactions for the *S-* and *R-*isomers of dihydrobenzofuran (A–D) and chromane (E, F) substituted PEAs in the 5-HT_2A_R. The receptor is shown as gray lines and cartoon, while ligands are displayed as sticks. Ligand–receptor interactions are displayed as dashed lines and colored in green (aromatic, π–π staking), yellow (hydrogen bond), and pink (salt-bridge).

As an attempt to investigate a hypothesis of a direct interaction from the *N*-benzyl moiety to Ser159^3×36^ as a determinant of bias and provide structural explanations for the experimental results, compounds **4a–4d** and **6a–6f** were docked (Figures 5 and 6) into the cryo-EM structure of the human 5-HT_2A_R bound to **6a** (25CN-NBOH) and coupled to a miniG_q_/G_i2_-G_s_ protein chimera.^52^ As validation of the docking protocol, the highest ranking binding pose of **6a** displayed a root-mean-square deviation (RMSD) of 0.57 Å for all heavy atoms, in comparison to the experimental ligand coordinates and reproduced all major ligand–receptor interactions, i.e., the canonical salt-bridge to the Asp155^3×32^, two hydrogen bonds to Ser159^3×36^, and aromatic interactions to Trp336^6×48^, Phe339^6×51^, and Phe340^6×52^ (Figure 5A).^52^ In general, the docking poses of the other compounds showed the same binding mode and interactions to the receptor as **6a**, with differences only in the pocket encompassing the *N*-benzyl moiety with differing substitution patterns (Figures 5A–G, 6A–F, and S7).

Focusing on Ser159^3×36^, only **6a** and **6b**, which both have oxygens in the ortho-position of the *N*-benzyl moiety (−OH or −OMe), establish the two hydrogen bonds to Ser159^3×36^ (Figure 5A,B). The fact that **6a** and **6b** display similar potencies in the two assays indicates that additional van der Waals interactions of the 2-methoxy of **6b** compensate for a weaker hydrogen bond relative to the 2-hydroxy substituent on the *N*-benzyl. While all other analogues, except **6c**, contain either a hydroxy group or an ether function, our docking poses of **4a**–**d** and **6d**–**f** do not show hydrogen bonds from the *N*-benzyl moiety to Ser159^3×36^ (Figures 5 and 6), corresponding to the lower potencies in both assays relative to **6a** and **6b** (Table 1). In fact, none of the three compounds that display significant bias (**4a**, **4b**, and **6e**—Figure 4) seem to form hydrogen bonds from the *N*-benzyl substituents to any of the surrounding receptor residues. The common trait between these three compounds is oxygen in the meta-position of the *N*-benzyl. While the docking poses showing placement of this functional group in a mainly hydrophobic receptor region (Figures 5b,c,g and S7) may explain the observed potency decreases (Table 1) compared to **6a** and **6b**, they do not provide a straightforward explanation for why the three compounds display bias. On the other hand, the meta-position points in the direction of Ser162^3×39^ and Ser373^7×46^, which could potentially change conformation when the ligands bind (induced-fit), something that the employed docking protocol does not account for. The substitution pattern of the *N*-benzyl may also impact the distribution of electron density of this aromatic ring and, thus, affect the interactions with the surrounding aromatic residues (Figures 5 and 6). Additionally, previous work has shown Leu362^7×34^ and Tyr370^7×42^ to play a role in bias.^43,53^ However, Leu362^7×34^ is outside of contact distance for all docked compounds and while Tyr370^7×42^ does display aromatic interactions to the *N*-benzyl of some compounds, this is not consistent with whether they show bias or not, e.g., **4b** but not **4a** displays aromatic contact to Tyr370^7×42^ (Figure 5B,C).

While the importance of direct hydrogen bonds between **6a** and Ser159^3×36^ is supported by marked and similar drops in both β-arr2 and miniGα_q_ potencies (14- and 16-fold) and efficacies (36 and 53%) in the S159A-mutated 5-HT_2A_R, the effect on the β-factor is remarkably subtle (0.619 vs 0.733, Tables 1 and 2). Since Ser159^3×36^ interacts with both the ammonium and the *N*-benzyl *ortho*-OH of **6a**, we cannot distinguish the influence of these two hydrogen bonds on the observed agonist potency and efficacy decreases. Regardless, this residue has little influence on β-arr2 vs miniGα_q_ bias for **6a**. Serotonin signaling is also markedly decreased by the S159A mutation in both assays (55-fold and 33% in β-arr2 plus 13-fold and 173% in miniGα_q_), indicating that the removal of the interaction between Ser159^3×36^ and the protonated amine (Figure S8) is the root cause for this decrease. The differential effects by the S159A-mutated 5-HT_2A_R on the two pathways observed for serotonin and the other compounds (excluding **6a**) may then be due to the lack of the additional hydrogen bond to Ser159^3×36^ seen in **6a**, which displays similar decreases. However, the effect of the S159A mutation on the β-factor for serotonin is again very small (0.576 vs 0.550—Tables 1 and 2). The only compound for which we have data showing a marked change in bias by the S159A mutation is **4b**, where the β-factor changes from 1.100 to 0.565. This does indicate that Ser159^3×36^ in combination with the *N*-benzyl substitution pattern in fact influences bias between β-arr2 and miniGα_q_, but apparently not via a direct hydrogen bond to the *N*-benzyl substituent. A water-bridged interaction could potentially play a role, but such an analysis cannot be performed with the docking protocol used here.

Regardless of the docking failing to provide a detailed explanation for the differing β-factors and the fact that most compounds do not display bias between β-arr2 and miniGα_q_, we can clearly see that the different *N*-benzyl substituents result in differential functional profiles in the β-arr2 and miniGα_q_ assays. Regarding **6b** as a reference (as it has similar potency and efficacy in the two assays), both potency and efficacy are in general higher in the β-arr2 vs the miniGα_q_ assay (Figure S9). Keeping in mind that the highest difference in potency between the two assays (for **6f**) only corresponds to a 6-fold change, this still indicates that the changes we made in the *N*-benzyl substitution in general have larger effects in the miniGα_q_ vs the β-arr2 assay, reflected in either potency and/or efficacy decrease. This demonstrates that alterations in the *N*-benzyl substitution pattern may be used to affect the preference between the two signaling pathways.

## Conclusions

In summary, 10 5-HT_2A_R ligands, based on the 5-HT_2A_R selective agonist **6a** (25CN-NBOH), were successfully designed and synthesized, with the aims of delineating their functional selectivity profiles in assays for Gq- and βarr2-mediated 5-HT_2A_R signaling and to evaluate the role of the hydrogen interaction of **6a** with Ser159^3×36^ in the receptor. The ligands were functionally characterized at 5-HT_2A_R in the βarr2- and miniGα_q_-recruitment assays. Compounds **4a–d**, **6c**, and **6e–f** lacked the possibility for simultaneous interaction of the ammonium and the *ortho*-oxygen on the benzyl moiety with Ser159^3×36^. The lack of interaction between the hydroxy and Ser159^3×36^ resulted in detrimental effects for both potency and efficacy, as assessed by βarr2 and miniGα_q_ recruitment assays. Remarkably, G_q_-mediated signaling was considerably more affected by the compounds’ lack of the ortho-hydrogen bond acceptor. The exact reasons for this observation could not be identified computationally, as the precise effect of the interaction of the benzylic hydroxyl and the interaction of the ammonium with Ser159^3×36^ could not be distinguished.

Regardless of the docking not being able to provide a detailed explanation for the differing β-factors and the fact that most compounds do not display bias between βarr2 and miniGα_q_, we can clearly see that the different *N*-benzyl substituents result in differential functional profiles in the βarr2 and miniGα_q_ assays. Keeping in mind that the highest difference in potency between the two assays (for **6f**) only corresponds to a 6-fold change, this still indicates that the changes we made in the *N*-benzyl substitution in general have larger effects in the miniGα_q_ vs the βarr2 assay, reflected in either potency and/or efficacy decrease. This demonstrates that alterations in the *N*-benzyl substitution pattern can be used to affect the preference between the two signaling pathways. Overall, these insights led to the development of **4a**-**b** and **6e**-**f**, the first efficacious 5-HT_2A_R agonists to be βarr2-biased, relative to LSD. Of special highlight is compound **4a** with potency and efficacy of 11.1 nM and 112%, respectively, for βarr2 recruitment, while in the miniGα_q_-recruitment assay, **4a** had potency and efficacy of 48.8 nM and 28.0%, respectively, as referenced by LSD. Compound **(±)–6f** showed potency and efficacy of 108 nM and 82.9%, respectively, for βarr2, while in the miniGα_q_-recruitment assay, compound **(±)–6f** exhibited potency and efficacy of 631 nM and 18.0%, respectively, as referenced by LSD. Therefore, **4a** and **6f** are interesting tool compounds to use for further evaluation of the role of signaling bias at the 5-HT_2A_R.

## Experimental Section

### Organic Chemistry

All reactions involving dry solvents or sensitive agents were performed under a nitrogen atmosphere and glassware was dried prior to use. Commercially available chemicals were used without further purification. Solvents were dried prior to use with an SG water solvent purification system or dried by standard procedures, and reactions were monitored by analytical thin-layer chromatography (TLC, Merck silica gel 60 F_254_ aluminum sheets). Flash chromatography was carried out using Merck silica gel 60A (35–70 *μ*m). ^1^H NMR spectra were recorded on a 400 MHz Bruker Avance III or 600 MHz Bruker Avance III HD, and ^13^C NMR spectra on a 101 MHz Bruker Avance III or 151 MHz Bruker Avance III HD. Analytical high-performance liquid chromatography (HPLC) was performed using an UltiMate HPLC system consisting of an LPG-3400A pump (1 mL/min), a WPS-3000SL autosampler, and a 3000 Diode Array Detector installed with a Gemini-NX C18 (250 mm × 4.60 mm, 3 μm) column. Solvent A: H_2_O + 0.1% trifluoroacetic acid (TFA); Solvent B: MeCN-H_2_O 9:1 + 0.1% TFA. For HPLC control, data collection, and data handling, Chromeleon software v. 6.80 was used. Ultrahigh-pressure liquid chromatography-mass spectrometry (UPLC-MS) spectra were recorded using an Acquity UPLC H-Class Waters series solvent delivery system equipped with an autoinjector coupled to an Acquity QDa and TUV detectors installed with an Acquity UPLCBEH C18 (50 mm × 2.1 mm, 1.7 μm) column. Solvent A: 5% aq MeCN + 0.1% HCO_2_H: Solvent B: MeCN + 0.1% HCO_2_H. Usually, gradients from A:B 1:0 to 1:1 (5 min) or A:B 1:0 to 0–50 (5 min) were performed depending on the polarity of the compounds. For data collection and data handling, MassLynx software was used. Optical rotations were determined in a thermostated cuvette on an Anton Paar MCP300 Modular Circular Polarimeter. Compounds were dried under high vacuum or freeze-dried using a ScanVac Cool Safe Freeze Drier. The purity of compounds submitted for pharmacological characterization was determined to be >95%, by HPLC analysis.

### General Procedure (A) for the Synthesis of Secondary Amines

The aldehyde (1.1 equiv) was added to a suspension of the phenethylamine hydrochloride (1 equiv) and Et_3_N (1.0 equiv) in EtOH. The reaction mixture was stirred until the formation of the imine was complete (30 min—3 h). After the addition of NaBH_4_ (2.0 equiv), the mixture was stirred for 45 min and concentrated under reduced pressure. The residue was partitioned in CH_2_Cl_2_/H_2_O (1:1 v/v), and the aqueous phase was further extracted with CH_2_Cl_2_ (2×). The organic layers were combined, dried over NaSO_4_, filtered, and evaporated under reduced pressure. The secondary amine product was purified by column chromatography (CH_2_Cl_2_/MeOH/Et_3_N, 98.2:1.4 + 0.24%) and precipitated by the addition of 4 M HCl in dioxane (1.5 equiv) under continuous stirring. The solid was filtered, dried under reduced pressure, dissolved in a minimum amount of MeOH, and precipitated by the addition of Et_2_O. The product was collected by filtration and dried under high vacuum.

### General Procedure (B) for the Synthesis of Conformational Constrained Derivatives

Glacial acetic acid (3.0 equiv) was added to a suspension of the targeted amine hydrochloride (1.0 equiv) in methanol/THF (2:1 v/v). 4-Chromanone (2.5 equiv) or 3-coumaranone (3 equiv) was added, and the reaction mixture was stirred at room temperature until the formation of the corresponding imine was complete based on TLC (CH_2_Cl_2_/MeOH/Et_3_N, 98.2:1.4 + 0.24%). NaBH_3_CN (in THF) (1.0 M, 3.0 equiv) was added and the reaction mixture was monitored by TLC and stirred for 30 min to 3 h. The mixture was quenched by the addition of NaHCO_3(aq)_, and the residue was extracted with EtOAc (3×). The combined organic extracts were dried over Na_2_SO_4_, filtered, and evaporated under reduced pressure. The secondary amine product was purified by column chromatography (CH_2_Cl_2_/MeOH/Et_3_N, 98.2:1.4 + 0.4%) and precipitated by the addition of 4 M HCl in dioxane (1.5 equiv) under continuous stirring. The solid was filtered, dried under reduced pressure, dissolved in a minimum amount of MeOH, and precipitated by the addition of Et_2_O. The product was collected by filtration and dried under high vacuum.

#### 2-(2,5-Dimethoxyphenyl)ethan-1-amine Hydrochloride (**2**)

The title compound was prepared according to reported conditions.^10^ Characterization was in accordance with reported values.^54^

#### 2-(4-Bromo-2,5-dimethoxyphenyl)ethan-1-amine Hydrochloride (**3**)

The title compound was prepared according to reported conditions.^10^ Characterization was in accordance with reported values.^54^

#### 3-(((4-Bromo-2,5-dimethoxyphenethyl)amino)methyl)phenol Hydrochloride (**4a**)

The title compound was prepared according to General procedure A and in line with reported conditions, and the characterization was in accordance with reported values.^45^

#### 2-(4-Bromo-2,5-dimethoxyphenyl)-*N*-(3-methoxybenzyl)ethan-1-amine Hydrochloride (**4b**)

The title compound was prepared according to General procedure A and in line with reported conditions, and the characterization was in accordance with reported values.^45^

#### (±)-*N*-(4-Bromo-2,5-dimethoxyphenethyl)-2,3-dihydrobenzofuran-3-amine Hydrochloride (**4c**)

The title compound was prepared according to General procedure B, which yielded the desired compound as a white solid in 52%. LCMS (ESI) *m*/*z* = 378.1 [M + H]^+^; ^1^H NMR (600 MHz, DMSO) δ 9.28 (s, 2H), 7.64 (d, *J* = 7.5 Hz, 1H), 7.38 (td, *J* = 7.8, 1.4 Hz, 1H), 7.22 (s, 1H), 7.04–6.99 (m, 2H), 6.99–6.96 (m, 1H), 5.10 (s, 1H), 4.75 (dd, *J* = 11.5, 2.7 Hz, 1H), 4.65 (dd, *J* = 11.4, 7.9 Hz, 1H), 3.80 (s, 4H), 3.76 (s, 4H), 3.14 (s, 2H), 2.95–2.84 (m, 2H). ^13^C NMR (151 MHz, DMSO) δ 160.7, 151.5, 149.4, 131.7, 127.2, 125.1, 121.2, 121.0, 115.9, 115.1, 110.4, 109.1, 58.0, 56.7, 56.3, 56.2, 43.5, 26.7.

#### (±)-*N*-(4-Bromo-2,5-dimethoxyphenethyl)chroman-4-amine Hydrochloride (**4d**)

The title compound was prepared according to General procedure B, which yielded the desired compound as a white solid in 58%. LCMS (ESI) *m*/*z* = 392.1 [M + H]^+^; ^1^H NMR (600 MHz, DMSO) δ 9.06 (s, 2H), 7.51 (d, *J* = 7.5 Hz, 1H), 7.32 (t, *J* = 7.7 Hz, 1H), 7.23 (s, 1H), 7.02 (s, 1H), 6.98 (t, *J* = 7.4 Hz, 1H), 6.89 (d, *J* = 8.2 Hz, 1H), 4.55 (s, 1H), 4.39–4.21 (m, 2H), 3.81 (s, 3H), 3.78 (s, 3H), 3.28–3.13 (m, 2H), 2.97 (dtd, *J* = 44.2, 12.4, 11.9, 5.4 Hz, 2H), 2.36–2.29 (m, 1H), 2.28–2.18 (m, 1H). ^13^C NMR (151 MHz, DMSO) δ 154.9, 151.5, 149.4, 130.7, 130.6, 125.3, 120.2, 117.2, 116.6, 115.9, 115.0, 109.1, 61.2, 56.7, 56.3, 50.2, 43.7, 26.5, 23.7.

#### 4-(2-Aminoethyl)-2,5-dimethoxybenzonitrile Hydrochloride (**5**)

The title compound was prepared according to reported conditions, and the characterization was in accordance with reported values.^46^

#### 4-(2-((2-Hydroxybenzyl)amino)ethyl)-2,5-dimethoxybenzonitrile Hydrochloride (**6a**)

The title compound was prepared according to reported conditions, and the characterization was in accordance with reported values.^32^

#### 2,5-Dimethoxy-4-(2-((2-methoxybenzyl)amino)ethyl)benzonitrile Hydrochloride (**6b**)

The title compound was prepared according to reported conditions, and the characterization was in accordance with reported values.^32^

#### 4-(2-((2-Fluorobenzyl)amino)ethyl)-2,5-dimethoxybenzonitrile Hydrochloride (**6c**)

The title compound was prepared according to reported conditions, and the characterization was in accordance with reported values.^32^

#### 4-(2-((Benzo[*d*][1,3]dioxol-4-ylmethyl)amino)ethyl)-2,5-dimethoxybenzonitrile Hydrochloride (**6d**)

The title compound was prepared according to reported conditions, and the characterization was in accordance with reported values.^32^

#### 4-(2-((3-Hydroxybenzyl)amino)ethyl)-2,5-dimethoxybenzonitrile Hydrochloride (**6e**)

The title compound was prepared according to General procedure A and in line with reported conditions, and the characterization was in accordance with reported values.^45^

#### (±)-4-(2-((2,3-Dihydrobenzofuran-3-yl)amino)ethyl)-2,5-dimethoxybenzonitrile Hydrochloride (**6f**)

The title compound was prepared according to General procedure B, which yielded the desired compound as a white solid in 55%. LCMS (ESI) *m*/*z* = 325.2 [M + H]^+^; ^1^H NMR (600 MHz, DMSO) δ 9.33 (s, 2H), 7.64 (d, *J* = 7.5 Hz, 1H), 7.38 (d, *J* = 6.2 Hz, 2H), 7.14 (s, 1H), 7.02 (t, *J* = 7.4 Hz, 1H), 6.98 (d, *J* = 8.1 Hz, 1H), 5.10 (s, 1H), 4.80–4.72 (m, 1H), 4.65 (dd, *J* = 11.4, 7.9 Hz, 1H), 3.88 (s, 3H), 3.79 (s, 3H), 3.18 (s, 2H), 3.04–2.91 (m, 2H). ^13^C NMR (151 MHz, DMSO) δ 160.7, 155.2, 150.9, 132.6, 131.7, 127.2, 121.0, 116.3, 115.0, 114.7, 110.4, 98.6, 72.3, 58.1, 56.6, 56.3, 43.2, 27.2.

### Pharmacology

#### Cell Culture and Transfection

The potency and efficacy of the synthesized substances are assessed by means of two distinct yet highly analogous bioassays, monitoring the recruitment of either β-arrestin 2 (βarr2) or miniGα_q_ to the activated target receptor (5-HT_2A_R). Essentially, the experimental procedures are carried out as described before, employing transiently transfected cells.^40−42^ Human embryonic kidney (HEK) 293T cells are maintained in Dulbecco’s modified Eagle’s medium (DMEM) (supplemented with GlutaMAX), containing 10% heat-inactivated fetal bovine serum (FBS), 100 IU/mL of penicillin, 100 μg/mL streptomycin, and 0.25 μg/mL amphotericin B. The cells are routinely cultured and incubated at 37 °C, in a humidified atmosphere containing 5% CO_2_. To quantify the activity of the ligands at the 5-HT_2A_R, the cells are transfected with the receptor construct (either the wild type or the S159A mutated 5-HT_2A_R fused to the LgBiT component of the NanoBiT system) and either SmBiT-βarr2 or SmBiT-miniGα_q_. To this end, the cells are seeded in six-well plates at a density of 500 000 cells per well. After 24 h, a transfection mixture is prepared consisting of a total of 3.3 μg of plasmid DNA and FuGENE HD transfection reagent, in a 3:1 FuGENE:DNA ratio, in OptiMEM I Reduced Serum Medium, incubated for 10 min, and added to the cells, according to the manufacturer’s protocol.

#### Assay Protocol

After overnight incubation of the transfected cells, the cells are reseeded in poly-d-lysine coated 96-well plates at a density of 50 000 cells per well. Following an additional 24 h incubation (in total, 48 h after transfection), the assay is started by rinsing the cells twice with 150 μL of Hank’s Balanced Salt Solution (HBSS) and adding 100 μL of HBSS to each well. To this, 25 μL of NanoGlo Live Cell Reagent is added (diluted 1/20 in LCS Dilution Buffer, according to the manufacturer’s protocol) and the plate is transferred to the Tristar2LB 942 multimode microplate reader (Berthold Technologies GmbH & Co, Germany), where the luminescent signal is measured during an equilibration phase. Upon signal stabilization, 10 μL of the 13.5 × concentrated agonist solutions is added to the wells—obtaining in-well concentrations of 25 μM–10 μM–1 μM–100 nM–10 nM–1 nM–100 pM–10 pM–1 pM, and the luminescence is monitored for 2 h. For each condition, the appropriate solvent controls are included. Each substance is tested in duplicate in at least three independent experiments, and reference substances LSD and serotonin are included in every experiment. For optimal comparability, the two assays are performed immediately after one another, using the same dilutions.

#### Cloning of the S159A-Mutated Receptor via Site-Directed Mutagenesis (SDM)

To assess the influence of residue S159 on the potency and efficacy of a selected subset of the substances, an S159A mutated 5-HT_2A_R was generated using a Phusion Site-Directed Mutagenesis kit, according to the manufacturer’s protocol. In brief, 200 pg of the template DNA (5-HT_2A_R-LgBiT) was mixed with the provided Phusion High Fidelity Mastermix and 0.5 μM of the forward primer (GTGCTCTTCGCCACGGCCTCCATCATGC) and reverse primer (GTCCAGGTAAATCCAGACTGCACAAAGCTTGC). The three-step polymerase chain reaction (PCR) was performed in a Mastercycler Nexus Thermal Cycler (Eppendorf, Hamburg, Germany) under the following conditions: initial denaturation (98 °C, 30 s), denaturation (98 °C, 10 s), annealing (71 °C, 20 s), extension (72 °C, 150 s), and final extension (72 °C, 5 min), of which the middle three steps were repeated 25 times. Following gel electrophoresis and purification, the linear product was religated with the provided T4 DNA ligase in the rapid ligation buffer and transformed into chemically competent *Escherichia coli* bacteria. After plasmid purification using the E.Z.N.A. Plasmid DNA Mini Kit (VWR International), the correctness of the construct was verified via Sanger sequencing.

#### Data Analysis

The resulting data were analyzed as described before in more detail.^55^ In brief, the obtained time–luminescence profiles are corrected for interwell variability and used for the calculation of the area under the curve (AUC), from which the AUC of the corresponding solvent control is subtracted. Data are then normalized using GraphPad Prism software (San Diego, CA), where the maximal response of the reference agonist is arbitrarily set at 100%. After pooling the data of the individual experiments, the potency and efficacy values are calculated in GraphPad Prism through three parametric nonlinear regression analysis. To quantify the tendency of the measured substances toward preferentially inducing one pathway or the other, bias factors are calculated via the “intrinsic relative activity approach.^56,57^ In this approach, an RA_i_ value is calculated for each substance in each of the measured assays, relative to a reference agonist, using the following formula
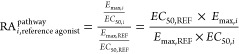
The obtained values for the respective pathways are then combined into a bias factor, β_i_
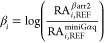
This formula implies that the value of β_i_ for the reference agonist is 0. A positive bias factor indicates a preference toward the recruitment of βarr2 over miniGα_q_, compared to the respective reference agonist. A negative bias factor then points to a relative preference toward the recruitment of miniGα_q_ over βarr2. To assess whether the obtained bias factors are statistically significant from 0, a Kruskal–Wallis analysis (which is the nonparametric counterpart of one-way analysis of variance (ANOVA), selected *a priori* to avoid presumptuous conclusions) with post hoc Dunn’s multiple comparison was carried out in GraphPad Prism. To qualitatively visualize the possible preference of a certain substance towards recruiting either one cytosolic protein or the other, bias plots were generated via GraphPad Prism. To this end, the normalized AUC values obtained in the βarr2 assay are plotted on the *x*-axis, and those obtained in the miniGα_q_ assay are plotted on the *y*-axis. On each plot, both the respective reference agonist and one substance of interest are plotted, and a curve is fitted through the centered second-order (quadratic) polynomial fitting.^58^

### Computational Methods

All molecular modeling calculations were performed in the Schrödinger Drug Discovery Suite (Release 2021–4, Schrödinger LLC, New York, NY, 2021). The ligands (**4a–d**, **6a–f**, and serotonin) were sketched in Maestro with the two-dimensional (2D) Sketcher tools, then the three-dimensional (3D) coordinates, charges, ionization states at pH 7.0 ± 2.0, and minimized conformations were generated with LigPrep using the default settings and the OPLS4 force field.^59^ For the ligands with multiple protonation states at physiological pH, only the state with a positive charge in the amino group (and a total charge of +1.0) was kept, as the salt-bridge interaction between the positive amine Asp155^3×32^ is crucial for ligand binding.^44^

The cryo-EM structure of 5-HT_2A_R bound to 25CN-NBOH and in complex to a mini-Gα_q_ protein chimera (accession code 6WHA)^52^ and the crystallographic structure of the LSD-bound 5-HT_2A_R (accession code 6WGT)^52^ were imported from PDB. For the cryo-EM structure, the coordinates of the G-protein and other auxiliary proteins were deleted, while for the crystallographic structure, only one protein chain (chain A) was kept. The 5-HT_2A_R structures were then prepared using Schrodinger’s Protein Preparation Wizard^60^ to add hydrogens, create disulfide bonds, generate protonation states for non-protein components using Epik v5.8^61^ at pH 7.0 ± 2.0, and complete missing side chains using Prime.^62,63^ For the bound ligands, 25CN-NBOH and LSD, the protonation state with the positive charge in the amine group was selected. The hydrogen-bond network of the protein was optimized with ProPKA^64,65^ at pH 7.0 and using ProtAssign^60^ to automatically optimize Asn, Gln, His, and hydroxyl side chains. This optimization was followed by two cycles of restrained minimization in the OPLS4 force field and with heavy atom convergence RMSD of 0.30 Å for each cycle, using Impact v9.3.^66^

The prepared 5-HT_2A_R structures were used to generate the docking grids. The grids were centered around the experimental ligand (25CN-NBOH or LSD), with no van der Waals scaling factor applied to receptor atoms. The side chains of Ser159^3×36^ Thr160^3×37^, Ser239^5×44^, Ser242^5×46^, and Tyr370^7×42^ were allowed to rotate. No additional constraints were applied, and other settings were kept in default values. The ligands **4a–d** and **6a–f** were docked in the cryo-EM structure of 5-HT_2A_R bound to 25CN-NBOH, while serotonin was docked in the crystallographic structure of the LSD-bound 5-HT_2A_R. The dockings were performed in Glide v9.3^67,68^ in extra precision mode and the OPLS4 force field.^69^ The van der Waals radii of ligand atoms were not scaled, as the docking involved a congeneric series to the experimental ligand. The sampling of nitrogen inversions, ring conformations, and the use of enhanced planarity for conjugated π groups was allowed. Five docking poses were written per ligand, followed by a post-docking optimization with a rejection threshold of 0.50 kcal/mol with the application of strain correction. All other settings were kept in the default values, while docking poses were selected based on the lowest docking score and lowest RMSD to the experimentally bound ligand.

Ligand–receptor interaction and structural interaction fingerprints (SIFt) were calculated with the Pymol plugin Intermezzo (v1.2, Ochoa, et al., unpublished, available at http://mordred.bioc.cam.ac.uk/intermezzo), with a binding pocket definition comprising the residues within 5.0 Å of 25CN-NBOH (or LSD) in the docking template structure. PyMOL (The PyMOL Molecular Graphics System, Schrödinger LLC, New York, 2020) was also used to generate the figures. The GPCRdb numbering scheme was used to assign the generic residue numbers throughout the text and figures.^70^

## Supporting Information Available

Concentration–response curves of each individual compound, for both βarr2 and miniGα_q_; functional properties of the tested compounds, with serotonin as the reference agonist, the bias plots and Kruskal–Wallis analysis, with serotonin as the reference agonist; additional computational data and HPLC traces of a representative number of tested compounds (PDF)
CSV file of the docking models used in Figures 5 and 6 (CSV)
PDB file of the docking models used in Figure 5 (PDB)
PDB file of the docking models used in Figure 6 (PDB)

## Supplementary Material

jm2c00702_si_002.csv

jm2c00702_si_003.pdb

jm2c00702_si_004.pdb

## Abbreviations Used

5-HT_2A_R: serotonin 2A receptor
βarr2: β-arrestin 2
25CN-NBOH: 4-(2-((2-hydroxybenzyl)amino)ethyl)-2,5-dimethoxybenzonitrile
LSD: lysergic acid diethylamide
NBOMe: *N*-benzylphenethylamines
