# Supplementary material for: Discovery of β-Arrestin-Biased 25CN-NBOH-Derived 5-HT2A Receptor Agonists
Source: J Med Chem. 2022 Sep 13;65(18):12031–43. doi: 10.1021/acs.jmedchem.2c00702 (PMC9511481; doi:10.1021/acs.jmedchem.2c00702)
Supplement: Supplementary file 1 — jm2c00702_si_001.pdf [file jm2c00702_si_001.pdf]

Supporting Information

Discovery of β-Arrestin-Biased 25CN-NBOH-Derived 5-HT_{2A} Receptor Agonists

Christian B. M. Poulie[1,†], Eline Pottie[2,†], Icaro A. Simon[1], Kasper Harpsøe[1], Laura D'Andrea[1], Igor V. Komarov,[3] David E. Gloriam[1], Anders A. Jensen[1], Christophe P. Stove[2,*], Jesper L. Kristensen[1,*]

[1]*Department of Drug Design and Pharmacology, Faculty of Health and Medical Sciences, University of Copenhagen, Jagtvej 160, DK—2100 Copenhagen, Denmark*

[2]*Laboratory of Toxicology, Department of Bioanalysis, Faculty of Pharmaceutical Sciences, Ghent University, Campus Heymans, Ottergemsesteenweg 460, B-9000 Ghent, Belgium*

[3]*Enamine Ltd., Kyiv 02094, Ukraine.*

† *These authors contributed equally to this work.*

* *Authors to whom correspondence should be addressed.*

Corresponding Author

CPS: E-mail: Christophe.Stove@ugent.be

JLK: E-mail: Jesper.Kristensen@sund.ku.dk

Table of Contents:

Concentration-Response curves of individual compounds (referenced with LSD) 3

Concentration-Response curves of individual compounds (referenced with serotonin (5-HT))............. 4

Functional properties of the tested compounds, with serotonin (5-HT) as the reference agonist........... 5

Additional Computational Data... 9

HPLC traces..11

References..17

Concentration-Response curves of individual compounds (referenced with LSD)

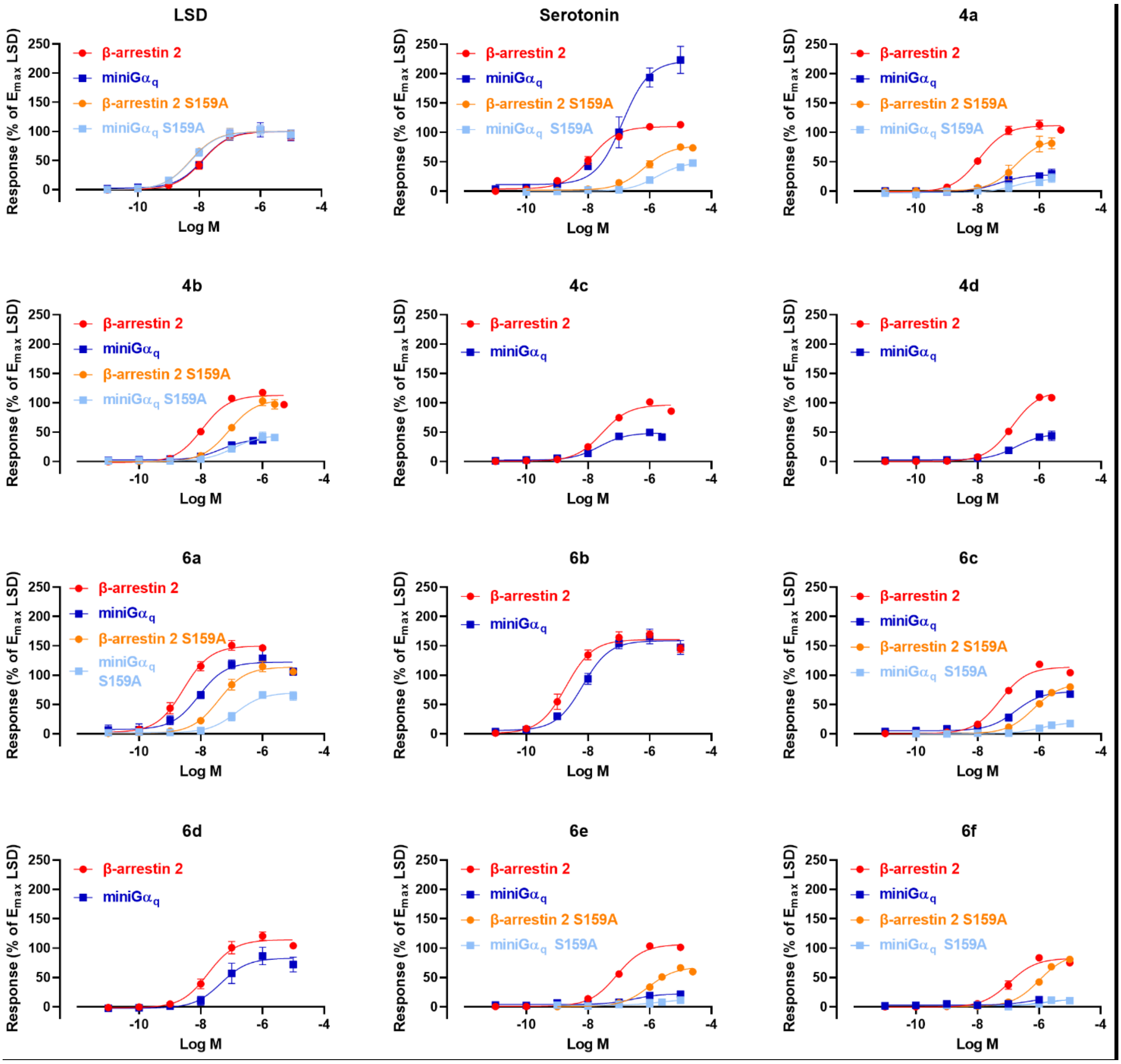

Figure S1. Concentration–response curves of the tested compounds (**4a–d**, and **6a–f**) in the 5-HT_{2A}R βarr2 and miniGα$_q$ recruitment assays, with LSD as a reference agonist and concentration–response curves of the tested compounds (**4a–b**, and **6a, c, e–f**) in the S159A mutated 5-HT_{2A}R βarr2 and miniGα$_q$ recruitment assays, with LSD as a reference agonist. Overlay of the concentration-response curves for each of the tested substances in the two assay formats. Each point represents the mean of three independent experiments, each performed in duplicate ± SEM (standard error of the mean). Curves represent three parametric, non-linear fits.

Concentration-Response curves of individual compounds (referenced with serotonin (5-HT))

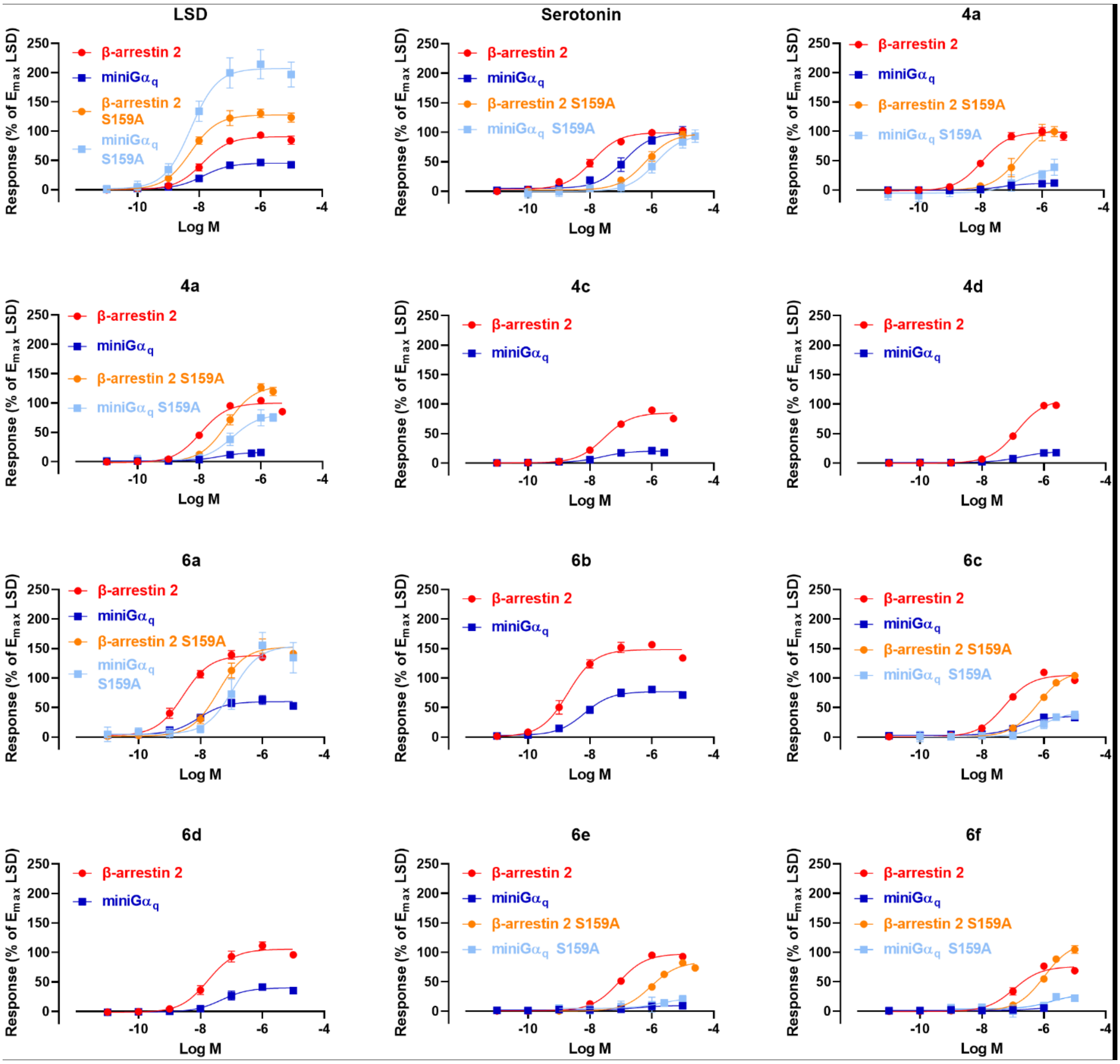

Figure S2. Concentration–response curves of the tested compounds (**4a–d**, and **6a–f**) in the 5-HT$_{2A}$R βarr2 and miniGα$_q$ recruitment assays, with serotonin as a reference agonist and concentration–response curves of the tested compounds (**4a–b**, and **6a, c, e–f**) in the S159A mutated 5-HT$_{2A}$R βarr2 and miniGα$_q$ recruitment assays, with serotonin as a reference agonist. Overlay of the concentration-response curves for each of the tested substances in the two assay formats. Each point represents the mean of three independent experiments, each performed in duplicate ± SEM (standard error of the mean). Curves represent three parametric, non-linear fits.

Functional properties of the tested compounds, with serotonin (5-HT) as the reference agonist

Table S1. Functional properties of the tested compounds (**4a–d**, and **6a–f**) on the 5-HT_{2A}R, and (**4a–b** and **6a, c, e–f**) on the S159A mutated 5-HT_{2A}R, in a βarr2 or miniGα_q recruitment assays, with serotonin as a reference agonist.[a]

**5-HT_{2A}**	**β-arr2**		**miniGα_q**		
	**EC_{50} (nM)<br>[CI]**	**E_{max} (%)<br>[CI]**	**EC_{50} (nM)<br>[CI]**	**E_{max} (%)<br>[CI]**	**β-factor**
**5-HT**	12.0<br>[8.41 - 17.1]	99.6<br>[95.1 – 104]	129<br>[64.9 – 258]	99.2<br>[88.4 – 111]	0
**LSD**	12.9<br>[8.45 - 19.9]	90.7<br>[85.0 - 96.4]	13.4<br>[6.82 - 26.5]	45.4<br>[41.1 - 49.9]	-0.718
**4a**	11.1<br>[7.45 - 16.4]	98.9<br>[92.8 – 105]	46.9<br>[11.9 – 158]	11.7<br>[9.10 - 14.6]	0.530
**4b**	11.1<br>[7.29 - 16.8]	100<br>[93.3 – 107]	43.3<br>[16.7 – 102]	16.2<br>[14.0 - 18.8]	0.386
**(±)-4c**	28.7<br>[19.5 - 42.4]	85.5<br>[80.2 - 90.9]	24.5<br>[12.1 - 50.1]	20.7<br>[18.6 - 22.9]	-0.431
**(±)-4d**	134<br>[111 – 161]	108<br>[104 – 113]	168<br>[78.7 – 402]	19.8<br>[16.8 - 23.7]	-0.152
**6a<br>(25CN-NBOH)**	2.76<br>[1.78 - 4.31]	139<br>[130 – 147]	8.66<br>[3.26 - 21.5]	60.1<br>[52.7 - 67.8]	-0.126
**6b<br>(25CN-NBOMe)**	1.94<br>[1.19 - 3.22]	148<br>[140 – 157]	6.53<br>[3.56 - 11.5]	77.1<br>[71.4 - 82.9]	-0.221
**6c<br>(25CN-NBF)**	53.3<br>[37.3 - 75.0]	105<br>[99.2 – 111]	164<br>[61.0 – 437]	35.3<br>[29.5 - 41.7]	-0.077
**6d<br>(25CN-NBMD)**	17.1<br>[10.5 - 28.0]	106<br>[97.9 – 114]	47.3<br>[17.7 – 117]	40.1<br>[33.4 - 47.3]	-0.107
**6e**	84.5<br>[64.0 – 111]	97.5<br>[92.9 – 102]	299<br>[43.9 - 1819]	9.75<br>[7.05 - 13.2]	0.548
**(±)-6f**	108<br>[68.6 – 169]	76.0<br>[69.5 - 82.6]	608<br>[n.d.]	7.72<br>[n.d.]	n.d.

**5-HT_{2A}-S159A**	**β-arr2**		**miniGα_q**		
	**EC_{50} (nM)<br>[CI]**	**E_{max} (%)<br>[CI]**	**EC_{50} (nM)<br>[CI]**	**E_{max} (%)<br>[CI)**	**β-factor**
**5-HT**	629<br>[385-995]	98.2<br>[90.6-106]	1317<br>[516-3745]	96.3<br>[79.5-118]	0
**LSD**	5.26<br>[3.21-8.44]	128<br>[120-136]	5.35<br>[2.32-11.7]	207<br>[186-229]	-0.533
**4a**	175<br>[82.3-411]	110<br>[94.4-130]	156<br>[n.d.]	36.8<br>[n.d.]	n.d.
**4b**	80.4<br>[55.1-116]	130<br>[121-139]	118<br>[44.3-332]	81.0<br>[67.5-97.1]	0.102
**6a<br>(25CN-NBOH)**	37.3<br>[22.9-59.9]	153<br>[141-165]	109<br>[39.5-279]	155<br>[126-185]	0.187
**6c<br>(25CN-NBF)**	645<br>[508-811]	113<br>[107-119]	899<br>[184-3332]	42.7<br>[29.3-63.7]	0.186
**6e**	938<br>[624-1364]	84.1<br>[77.4-91.2]	2060<br>[n.d.]	22.9<br>[n.d.]	n.d.
**(±)-6f**	1031<br>[695-1491]	118<br>[107-130]	1652<br>[n.d.]	28.9<br>[n.d.]	n.d.

[a] Data obtained in the βarr2 or miniGα_q recruitment assays, using the 2h time-luminescence profile to calculate the AUC. The EC_{50} value is a measure of agonist potency, and the E_{max} value is a measure of agonist efficacy. The E_{max} values for the compounds are normalized to E_{max} of serotonin as the reference agonist. Data are combined from at least three independent experiments, each performed in duplicate. The reported β-factor is the average value of the three β-factors obtained in three independent experiments. n.d. is not determined. CI: 95% confidence interval.

Table S2: Alternative method of calculation of the bias β-factor.[a]

5-HT$_{2A}$	Referenced with LSD		Referenced with 5-HT	
	β-factor	Alternative β-factor	β-factor	Alternative β-factor
**4a**	1.240	1.240	0.530	0.520
**4b**	1.100	1.060	0.386	0.348
**4c**	0.279	0.194	-0.431	-0.486
**4d**	0.558	0.516	-0.152	-0.198
**6a (25CN-NBOH)**	0.619	0.572	-0.126	-0.172
**6b (25CN-NBOMe)**	0.526	0.538	-0.221	-0.223
**6c (25CN-NBF)**	0.669	0.687	-0.077	-0.072
**6d**	0.638	0.553	-0.107	-0.169
**6e**	1.250	1.220	0.548	0.516
**6f**	n.d.	1.420	n.d.	0.711
**5-HT$_{2A}$-S159A**	**Referenced with LSD**		**Referenced with 5-HT**	
	**β-factor**	**Alternative β-factor**	**β-factor**	**Alternative β-factor**
**4a**	n.d.	0.566	n.d.	0.096
**4b**	0.565	0.494	0.102	0.043
**6a (25CN-NBOH)**	0.733	0.753	0.187	0.131
**6c (25CN-NBF)**	0.731	0.842	0.186	0.237
**6e**	n.d.	1.050	n.d.	0.577
**6f**	n.d.	1.010	n.d.	0.486

[a] Both β-factors are obtained via the formulas specified in the Materials and Methods section. The column 'β-factor' is the same as that reported in Table 1 and Table S1, and is the average of the β-factors calculated in each individual experiment. The column 'Alternative β-factor' is obtained by applying the same formulas on the 'combined' EC_{50} and E_{max} values, as reported in Table 1, 2 and Table S1. n.d. is not determined.

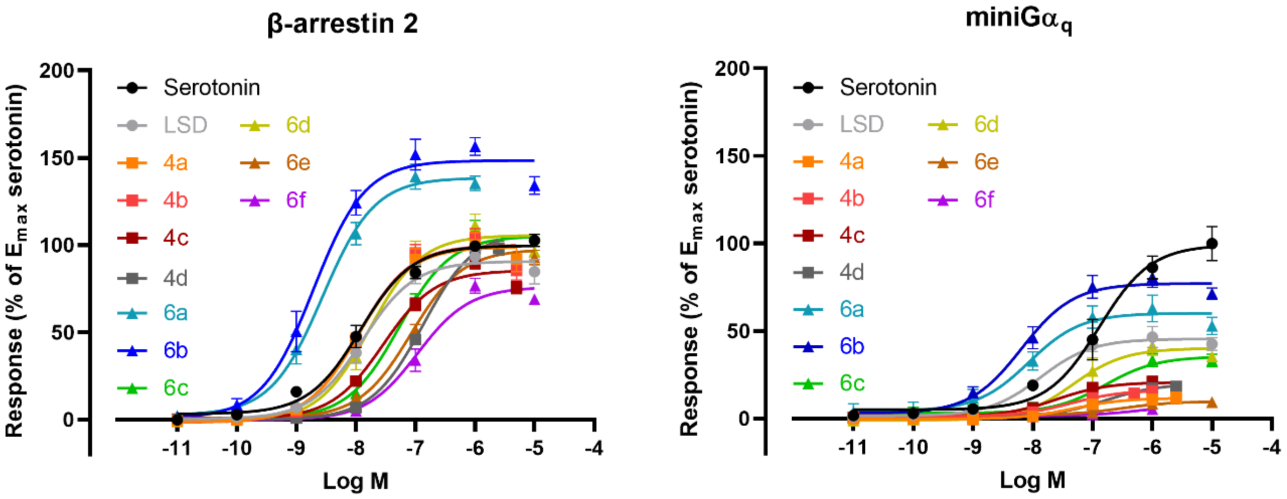

Figure S3. Concentration–response curves of the tested compounds (**4a–d**, and **6a–f**) in the 5-HT$_{2A}$R βarr2 or miniGα$_q$ recruitment assays, with serotonin as a reference agonist. Overlay of the concentration-response curves for each of the tested substances in the two assay formats. Each point represents the mean of three independent experiments, each performed in duplicate ± SEM (standard error of the mean). Curves represent three parametric, non-linear fits.

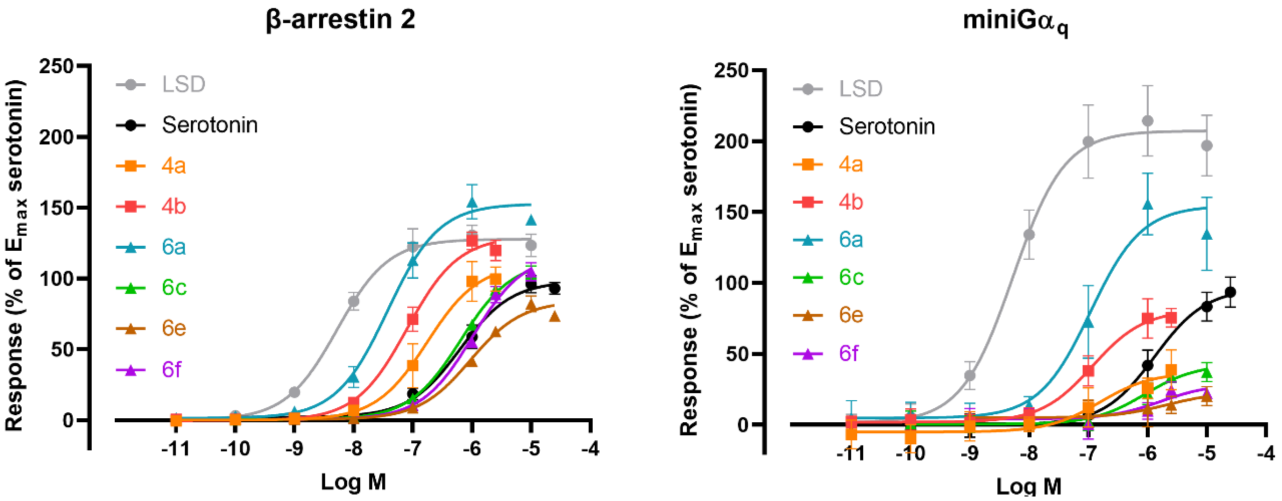

Figure S4. Concentration–response curves of the tested compounds (**4a–b**, and **6a, c, e–f**) in the S159A mutated 5-HT_{2A}R receptor βarr2 or miniGα_q recruitment assays, with serotonin as a reference agonist. Overlay of the concentration-response curves for each of the tested substances in the two assay formats. Each point represents the mean of three independent experiments, each performed in duplicate ± SEM (standard error of the mean). Curves represent three parametric, non-linear fits.

Bias plots and Kruskal-Wallis analysis, with serotonin (5-HT) as the reference agonist

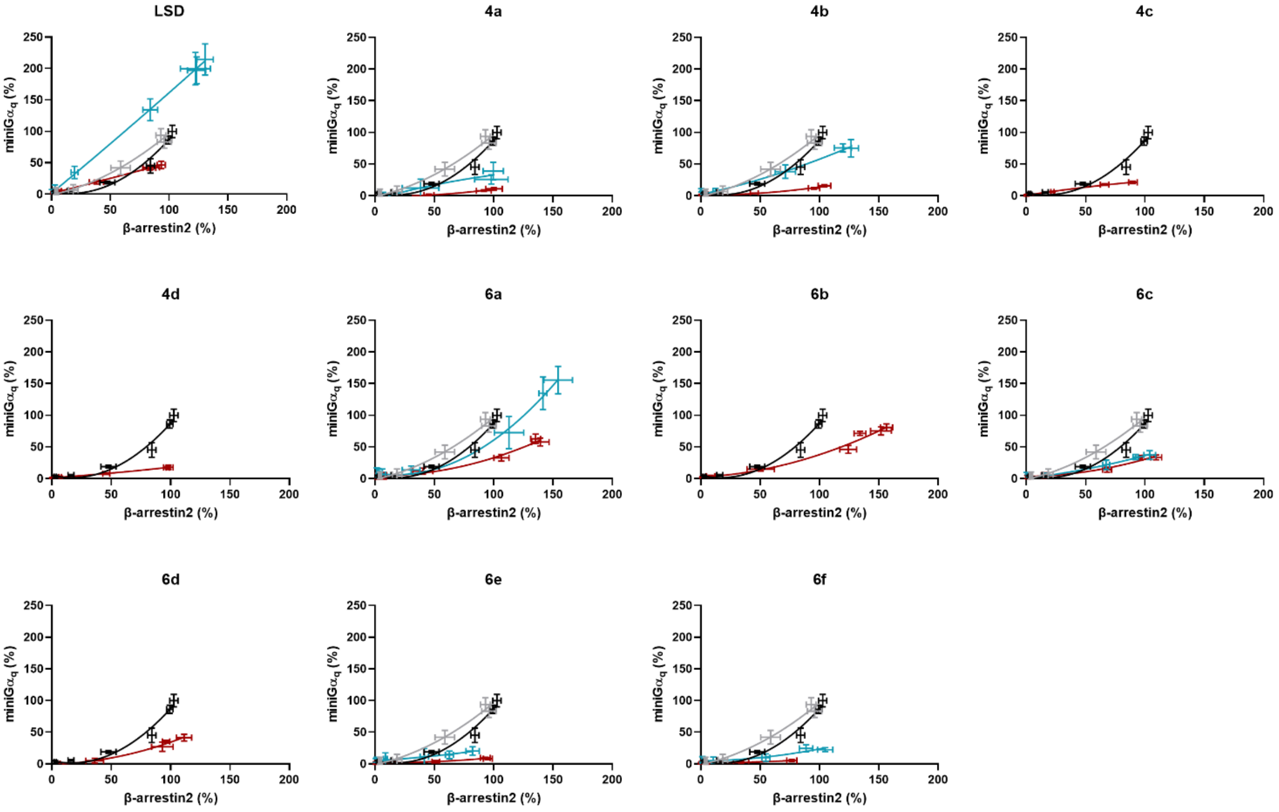

Figure S5. Qualitative bias plots, where each panel shows the centered second order polynomial fit of the activation values at equimolar concentrations of the substance in the respective assays in red, and that of the reference agonist (serotonin) in black for WT receptor and gray is reference (serotonin) data for the S159A mutated receptor. Red is data for WT receptor and blue is data for the S159A mutated receptor. Error bars represent the SEM of the individual data points per concentration.

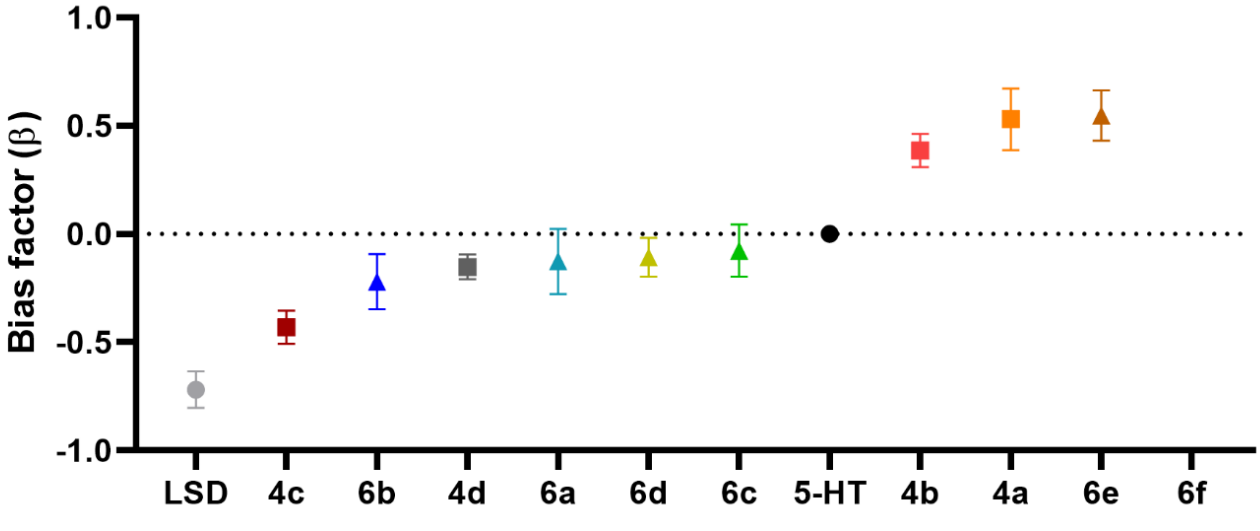

Figure S6. Visual representation of the bias factors (β) ± SEM, with serotonin as a reference agonist.

Additional Computational Data

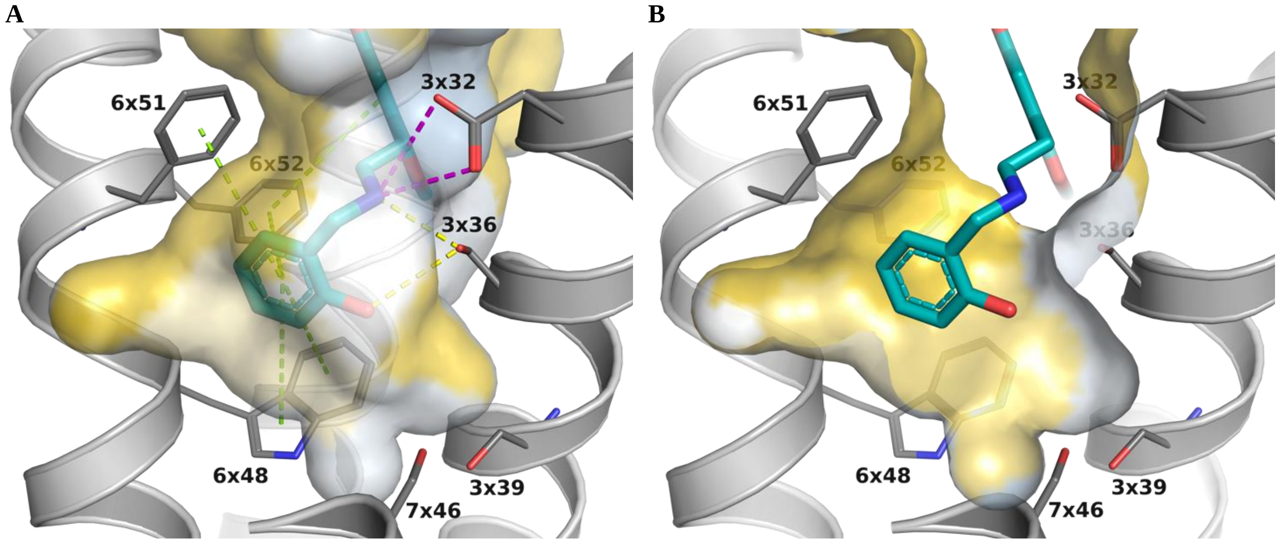

6a (25CN-NBOH) **6a** (25CN-NBOH)

Figure S7. *N*-benzyl rings of NBOMes occupy a distinctive subpocket in the 5-HT$_{2A}$ which mediates Gα_q protein signaling via hydrophilic interactions. Interactions between receptor (gray lines and cartoon) and the *N*-benzyl moiety of **6a** (25CN-NBOH) (cyan sticks) in the cryo-EM structure of the Gα_q-coupled 5-HT$_{2A}$ (PDB ID 6WHA),[1] with the *N*-benzyl subpocket displayed as a closed (A) surface and colored according to the Eisenberg hydrophobicity scale,[2] from highly hydrophilic (blue) to highly hydrophobic (yellow).

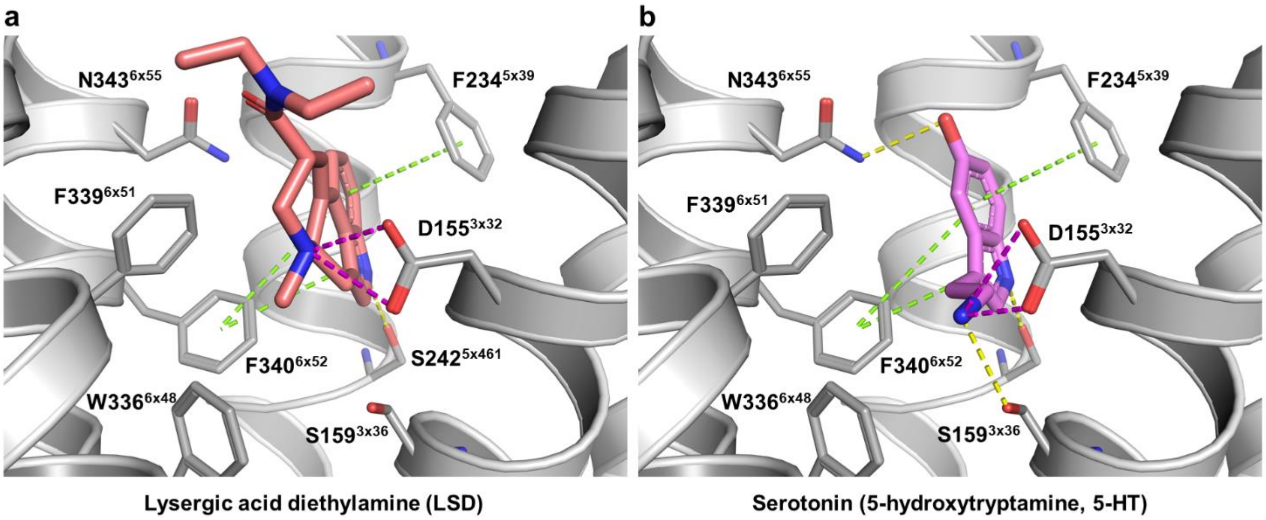

Figure S8. Experimental binding mode of LSD and predicted binding mode of serotonin at the 5-HT$_{2A}$ receptor reveal differential engagements to Ser159^{3x36}, reflected in the miniGα_q recruitment response. A. Interactions between receptor (gray lines and cartoon) and LSD (salmon sticks) in the crystallographic structure of the 5-HT$_{2A}$ (PDB ID 6WGT)[1]; B. Predicted binding pose of serotonin (pink sticks) and ligand-receptor interactions in the same structure. The interactions are displayed as dashed lines and colored in green (aromatic, π-π staking), yellow (hydrogen bond), and pink (salt-bridge).

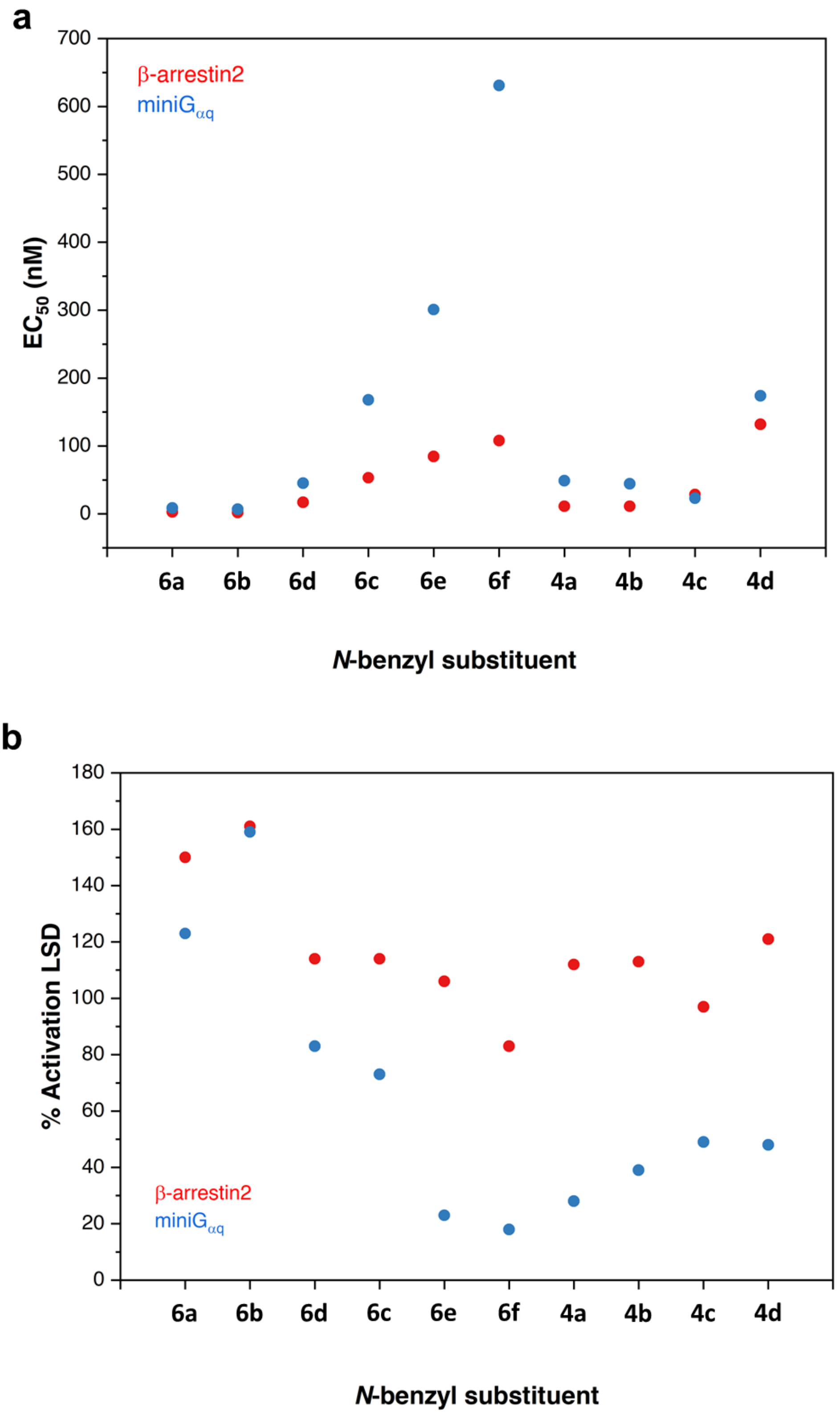

Figure S9. Interactions between the *N*-benzyl substituents and Ser159^{3x36} drive the preferential recruitment of Gα_q. (**A**) Potency (EC_{50}) and (**B**) efficacy (E_{max} relative to LSD) in the functional complementation assays for β-arrestin recruitment (red) and miniGα_q recruitment (blue) of the evaluated agonists.

HPLC traces

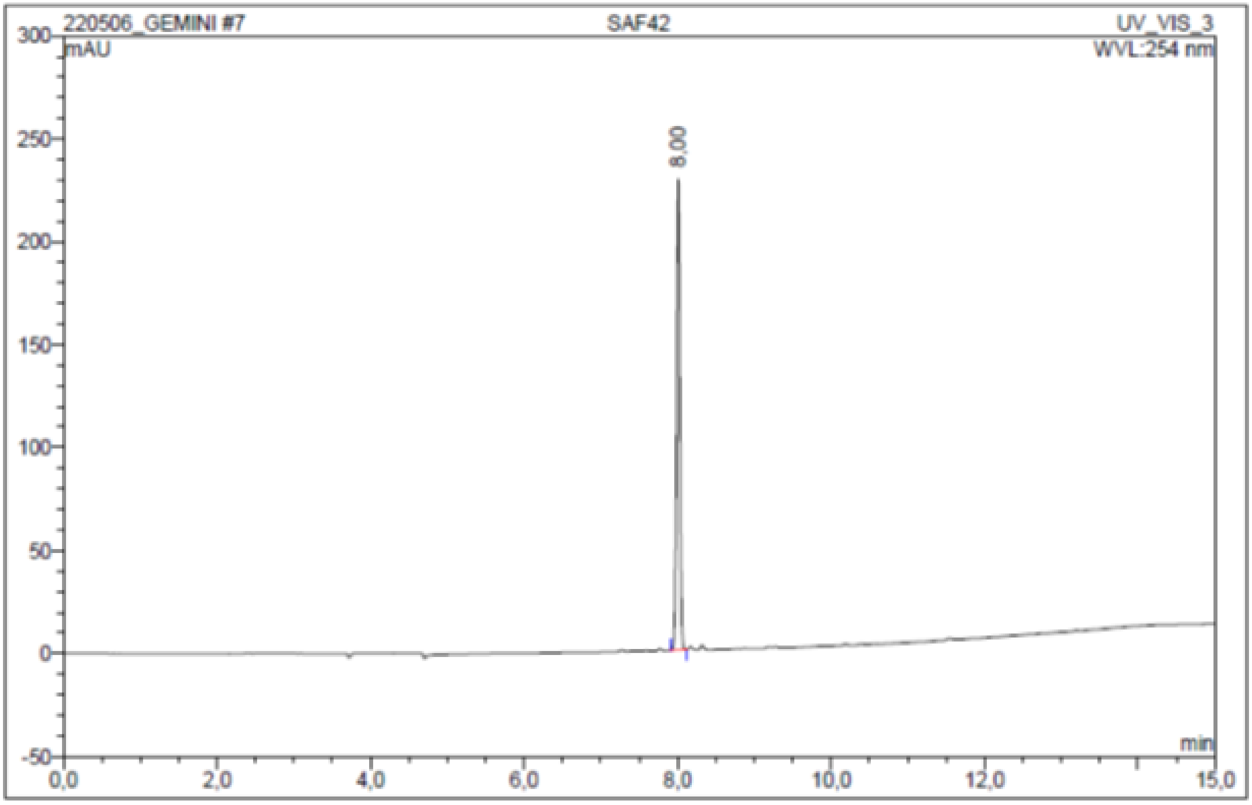

No.	Ret.Time min	Peak Name	Height mAU	Area mAU*min	Rel.Area %	Amount	Resolution(EP)
1	8,00	n.a.	228,896	10,694	100,00	n.a.	n.a.
Total:			228,896	10,694	100,00	0,000	

Figure S10. HPLC traces of compounds **4a**.

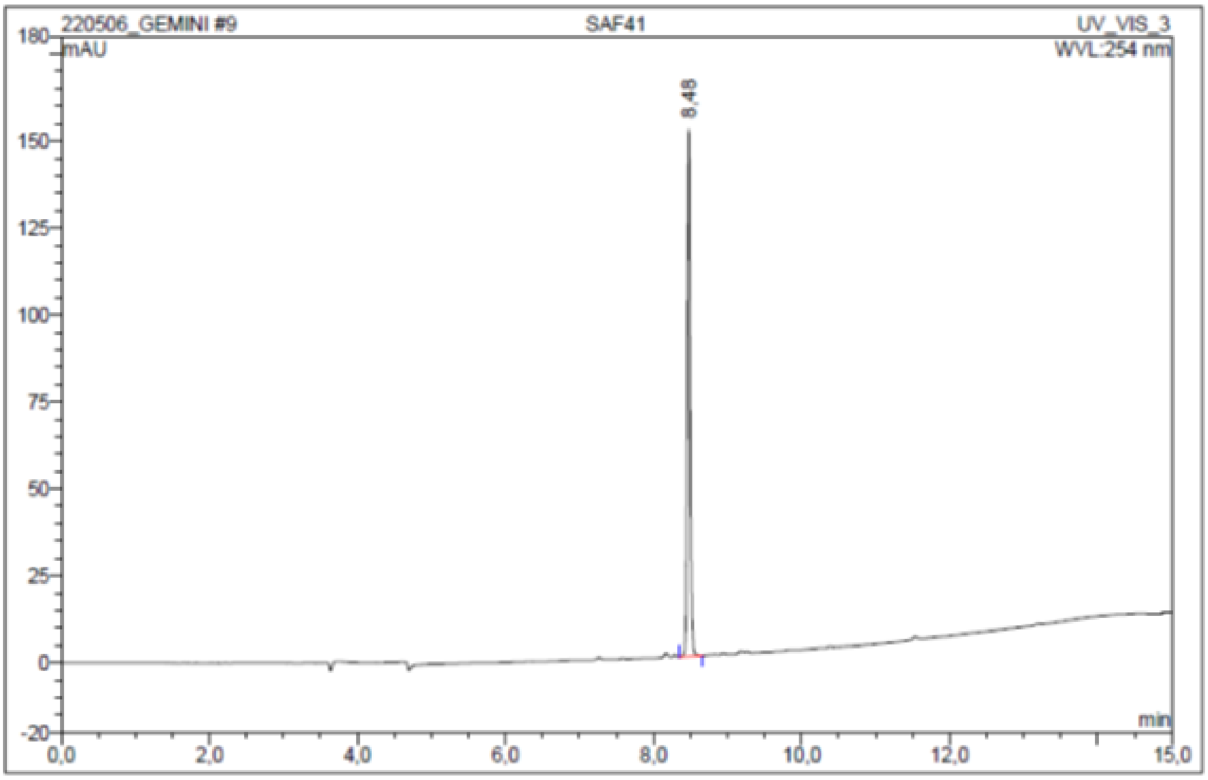

No.	Ret.Time min	Peak Name	Height mAU	Area mAU*min	Rel.Area %	Amount	Resolution(EP)
1	8,48	n.a.	151,474	7,020	100,00	n.a.	n.a.
Total:			151,474	7,020	100,00	0,000	

Figure S11. HPLC traces of compounds **4b**.

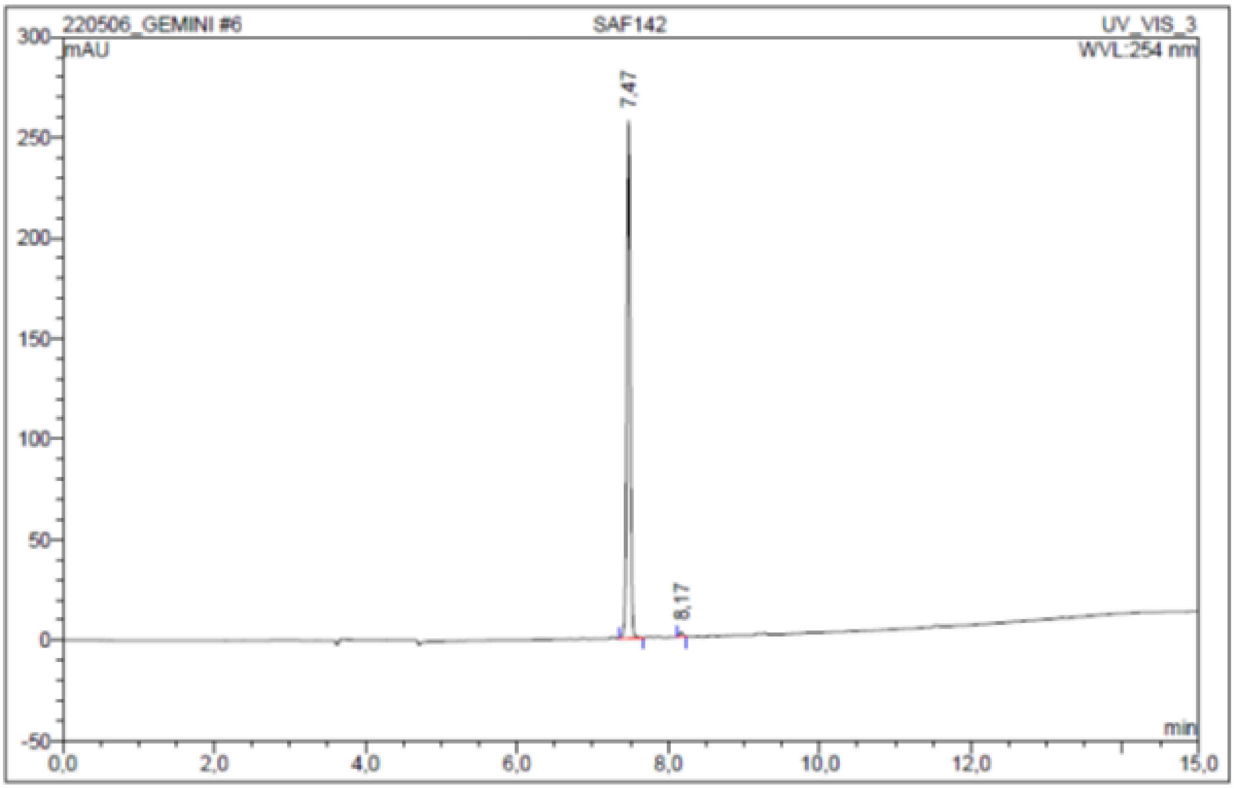

No.	Ret.Time min	Peak Name	Height mAU	Area mAU*min	Rel.Area %	Amount	Resolution(EP)
1	7,47	n.a.	257,553	13,240	99,28	n.a.	8,81
2	8,17	n.a.	1,980	0,097	0,72	n.a.	n.a.
Total:			259,533	13,336	100,00	0,000	

Figure S12. HPLC traces of compounds **4d**.

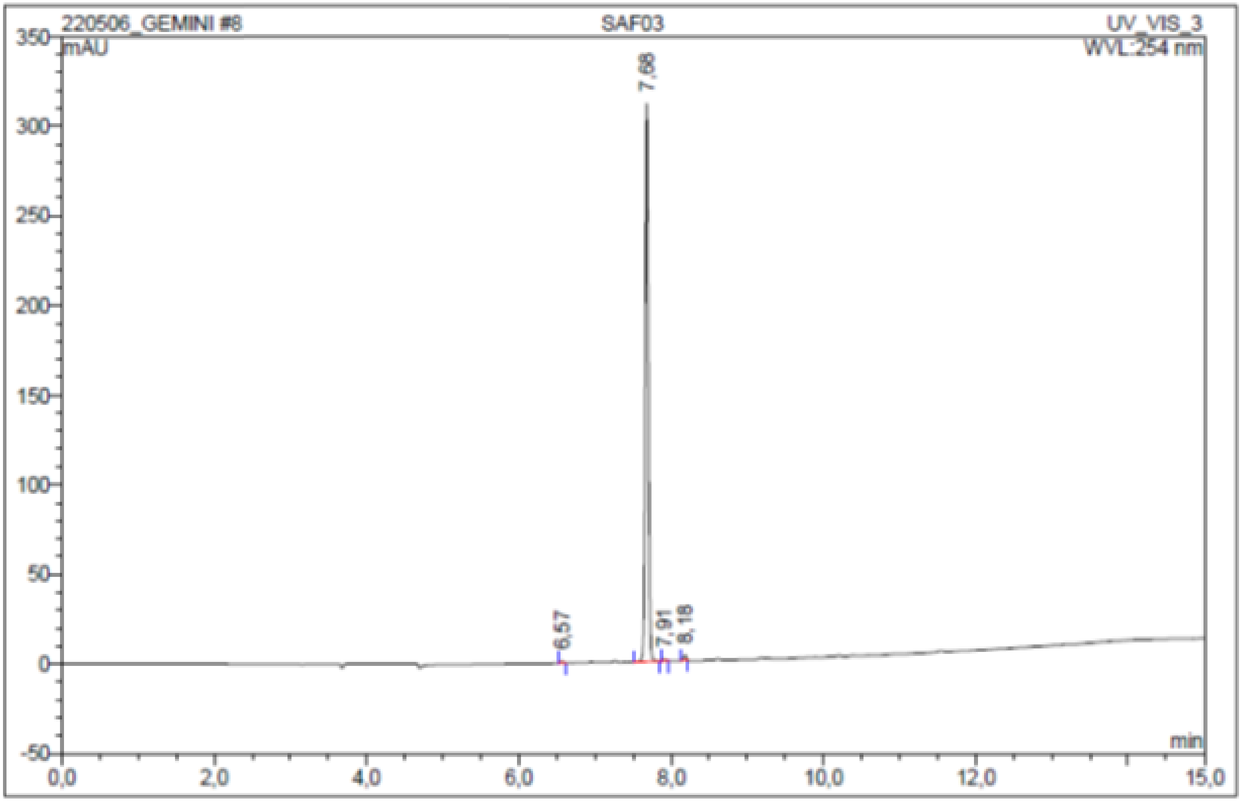

No.	Ret.Time min	Peak Name	Height mAU	Area mAU*min	Rel.Area %	Amount	Resolution(EP)
1	6,57	n.a.	0,962	0,041	0,27	n.a.	15,32
2	7,68	n.a.	311,409	15,040	98,64	n.a.	2,97
3	7,91	n.a.	0,910	0,046	0,30	n.a.	3,58
4	8,18	n.a.	2,686	0,121	0,79	n.a.	n.a.
Total:			315,967	15,248	100,00	0,000	

Figure S13. HPLC traces of compounds **6a**.

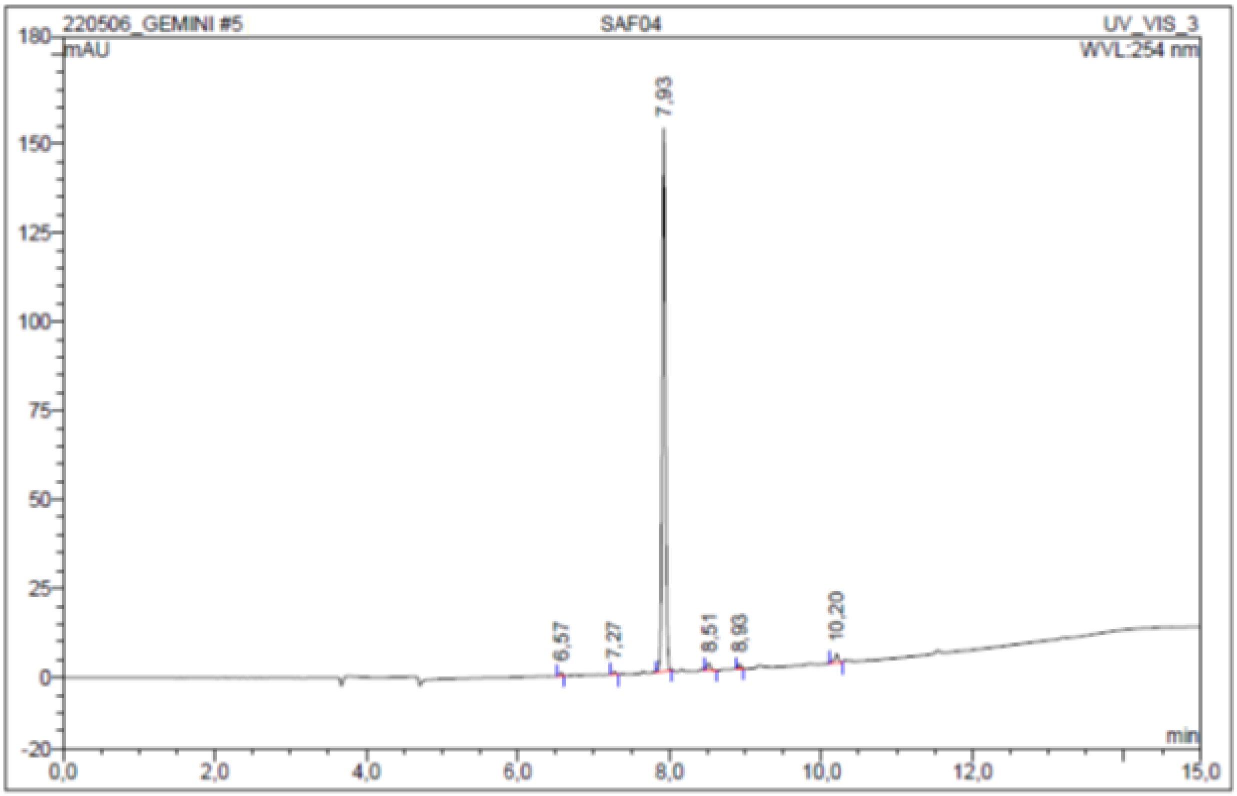

No.	Ret.Time min	Peak Name	Height mAU	Area mAU*min	Rel.Area %	Amount	Resolution(EP)
1	6,57	n.a.	0,831	0,037	0,48	n.a.	8,87
2	7,27	n.a.	0,672	0,034	0,44	n.a.	8,31
3	7,93	n.a.	152,573	7,343	95,40	n.a.	7,57
4	8,51	n.a.	1,892	0,096	1,25	n.a.	5,44
5	8,93	n.a.	1,241	0,054	0,71	n.a.	16,60
6	10,20	n.a.	2,301	0,133	1,73	n.a.	n.a.
Total:			159,511	7,697	100,00	0,000	

Figure S14. HPLC traces of compounds **6d**.

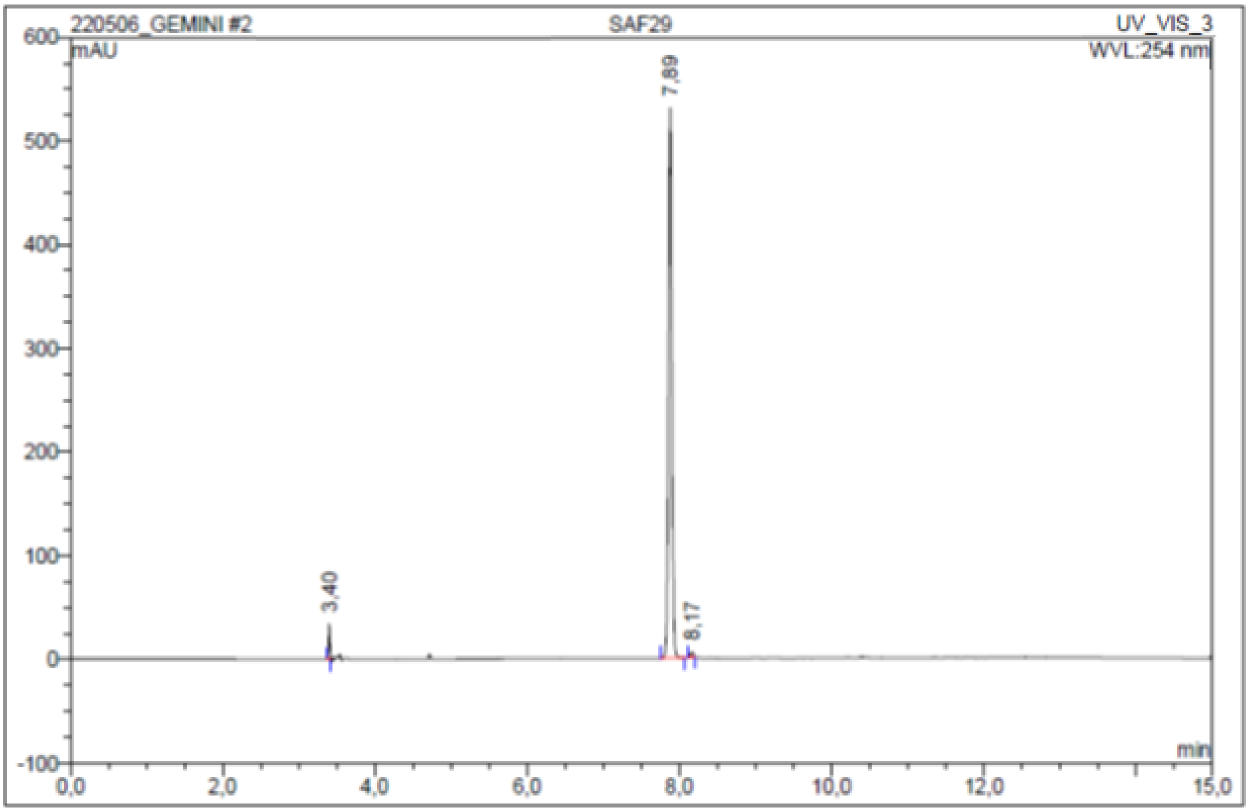

No.	Ret.Time min	Peak Name	Height mAU	Area mAU*min	Rel.Area %	Amount	Resolution(EP)
1	3,40	n.a.	33,908	0,755	2,50	n.a.	72,47
2	7,89	n.a.	530,574	29,205	96,87	n.a.	3,52
3	8,17	n.a.	4,364	0,190	0,63	n.a.	n.a.
Total:			568,846	30,150	100,00	0,000	

Figure S15. HPLC traces of compounds **6d**.

References

(1) Kim, K.; Che, T.; Panova, O.; DiBerto, J. F.; Lyu, J.; Krumm, B. E.; Wacker, D.; Robertson, M. J.; Seven, A. B.; Nichols, D. E.; Shoichet, B. K.; Skiniotis, G.; Roth, B. L. Structure of a Hallucinogen-Activated Gq-Coupled 5-HT2A Serotonin Receptor. *Cell* **2020**, *182* (6), 1574-1588.e19. https://doi.org/10.1016/j.cell.2020.08.024.

(2) Eisenberg, D.; Schwarz, E.; Komaromy, M.; Wall, R. Analysis of Membrane and Surface Protein Sequences with the Hydrophobic Moment Plot. *Journal of Molecular Biology* **1984**, *179* (1), 125–142. https://doi.org/10.1016/0022-2836(84)90309-7.
